# Preclinical B cell depletion and safety profile of a brain‐shuttled crystallizable fragment‐silenced CD20 antibody

**DOI:** 10.1002/ctm2.70178

**Published:** 2025-03-21

**Authors:** Vanessa L. Schumacher, Solen Pichereau, Juliana Bessa, Juergen Bachl, Sylvia Herter, Felix C. Weber, Johannes Auer, Anja Kipar, Michael Winter, Martina Stirn, Michael B. Otteneder, Kevin Brady, Anne Eichinger‐Chapelon, Adrian Roth, Nadine Stokar‐Regenscheit, Nicole Clemann, Shanon Seger, Claudia Senn, Juliane Hönig, Cordula Jany, Elisa Di Lenarda, Alain C. Tissot, Christian Klein, H.‐Christian von Büdingen, Robert Mader, Mohammed Ullah, Niels Janssen, Eduard Urich

**Affiliations:** ^1^ Roche Pharma Research and Early Development Roche Innovation Center Basel Switzerland; ^2^ Roche Pharma Research and Early Development Roche Innovation Center Zurich Switzerland; ^3^ Roche Pharma Research and Early Development Roche Innovation Center Munich Germany; ^4^ Laboratory for Animal Model Pathology, Institute of Veterinary Pathology Vetsuisse Faculty, University of Zurich Zürich Switzerland; ^5^ Department of Infection Biology and Microbiomes, Institute of Infection, Veterinary and Ecological Sciences University of Liverpool Liverpool UK; ^6^ Personalized Healthcare Safety, Product Development F. Hoffmann‐La Roche Ltd. Basel Switzerland; ^7^ Clinical Science, Neuroscience, Product Development F. Hoffmann‐La Roche Ltd. Basel Switzerland; ^8^ Present address: Debiopharm Lausanne Vaud Switzerland; ^9^ Present address: KB NBE Consulting, Charfield, Wotton‐under‐Edge UK; ^10^ Present address: Bayer AG Leverkusen 51373 Germany; ^11^ Present address: Curie.Bio, LLC Boston MA 02115 USA; ^12^ Present address: Ridgeline Discovery GmbH Technologiepark Hochbergerstrasse 60F, CH‐4057 Basel Switzerland; ^13^ Present address: Novartis Neuroscience Basel Switzerland

**Keywords:** antibodies, antigens, blood‐brain barrier, CD20, central nervous system, monoclonal, multiple sclerosis

## Abstract

**Background:**

The blood–brain barrier (BBB) presents a major challenge for the development of monoclonal antibody (mAb)‐based therapies for brain disorders. To improve the likelihood of success of such therapies, Roche Brainshuttle technology utilizes a single anti‐transferrin receptor 1 (TfR1)‐antigen‐binding antibody fragment linked to a therapeutic antibody, allowing engagement with TfR1 to transport the therapeutic antibody into the brain via receptor‐mediated transcytosis.

**Methods:**

We compared Fc‐silenced and Fc‐competent variants of the Brainshuttle and the parental (non‐shuttled) type II CD20 mAb, obinutuzumab in in vitro and in vivo (mouse and cynomolgus macaque) models. Endpoints assessed included B cell binding, B cell killing, tolerability, and ability to cross the BBB.

**Results:**

The Fc‐silenced Brainshuttle construct showed a superior safety profile compared with the Fc‐competent construct while maintaining the ability to cross the BBB and to deplete B cells in head‐to‐head comparisons in human and mouse in vitro and in mouse and cynomolgus macaque in vivo models.

**Conclusion:**

Together, our data provide a path forward for the future development of safe and efficacious brain‐targeted B‐cell‐depleting therapies.

**Key points:**

- The BBB hinders mAb‐based brain disorder therapies
- A brain‐targeted B‐cell‐depleting mAb for MS that efficiently crosses the BBB via hTfR1 was developed using Brainshuttle^™^ technology (1a and 1b)
- The Brainshuttle^™^‐CD20 mAb was well tolerated (2a and 2b) and displayed B‐cell‐killing properties (1c), paving the way for future development and clinical translation of TfR1‐targetingtherapies for increased brain penetration

## Graphical abstract

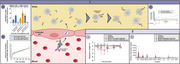

## 1 BACKGROUND

The blood–brain barrier (BBB) prevents neurotoxic plasma components, blood cells, and pathogens from entering the brain. Simultaneously, the BBB significantly impedes access to systemically administered monoclonal antibodies (mAbs) to the brain, limiting the delivery of therapeutically active mAbs and the ability to achieve therapeutic concentrations. This presents a major challenge in developing mAb‐based therapies for brain disorders. Approaches to cross the BBB through receptor‐mediated transcytosis of large molecules have therefore received significant attention. Receptors that are highly expressed at the BBB, where transcytosis has been well characterized, include transferrin receptor 1 (TfR1), low‐density lipoproteins, insulin, and leptin.^1^ In particular, TfR1, which is expressed on BBB endothelial cells, is an attractive target for enabling receptor‐mediated transcytosis of mAbs across the BBB and several approaches targeting this receptor have been previously published.^2^, ^3^, ^4^, ^5^, ^6^, ^7^, ^8^, ^9^, ^10^ Roche Brainshuttle* technology (*Brainshuttle is a registered trademark of F. Hoffmann‐La Roche) utilizes a single anti‐TfR1 antigen‐binding antibody fragment (Fab), referred to as the Brainshuttle module, fused to a cargo mAb for monovalent engagement of TfR1 and transport into the brain.^3^, ^11^ Here we describe the application of Brainshuttle technology and clinical candidate selection of a brain‐targeted B‐cell‐depleting mAb for the treatment of multiple sclerosis (MS).

MS is a chronic, inflammatory, demyelinating, and degenerative neurological disease.^12^ Historically, it was believed that central nervous system (CNS) tissue damage in MS was mediated by infiltrating proinflammatory CD4+ T cells.^13^, ^14^ However, it is now known that B cells are key contributors to MS immunopathology, as demonstrated by the observed clinical efficacy of CD20‐targeting, B‐cell‐depleting therapies in relapsing MS.^15^, ^16^, ^17^, ^18^, ^19^, ^20^, ^21^, ^22^, ^23^ Ocrelizumab, the first anti‐CD20 mAb approved for the treatment of MS, has shown substantial efficacy on the annualized relapse rate and a moderate efficacy on disability progression in individuals with MS.^24^ While ocrelizumab mainly targets peripheral CD20‐expressing B cells, a growing body of evidence suggests that meningeal inflammation containing CD20+ B cells and CNS‐compartmentalized B cells are associated with gray matter pathology and clinical progression in MS.^25^, ^26^ Efficient depletion of CNS‐compartmentalized and meningeal B cells, in addition to peripheral B cells, might lead to further reduction of progressive CNS tissue damage in MS, and with that, further reduce the risk of disability progression in those who suffer with MS.

CD20 is a protein expressed on the surface of B cells and is a clinically validated MS therapeutic target.^27^ There are two classes of CD20 mAbs: type I antibodies such as rituximab, ocrelizumab, or ofatumumab, and type II antibodies like tositumomab or obinutuzumab.^28^, ^29^ Obinutuzumab is differentiated from rituximab and other type I antibodies by virtue of mediating stronger direct B cell death, independent of crystallizable fragment (Fc) effector functions, and enhanced antibody‐dependent cell‐mediated cytotoxicity/antibody‐dependent cellular phagocytosis (ADCC/ADCP) due to Fc‐engineering, but reduced complement‐dependent cytotoxicity (CDC).^30^, ^31^, ^32^ Type I CD20 binding leads to CD20 clustering, by bridging CD20 dimers due to the binding of the two Fabs within a CD20 dimer,^33^ which induces good complement activation, whereas type II binding with one single CD20 dimer shows more potent ADCC and induction of direct cellular cytotoxicity.^28^, ^34^, ^35^ In order to most efficiently target CNS‐compartmentalized B cells in MS, a CD20 mAb would need to possess properties for extravascular B cell depletion and the ability to cross the BBB. We reasoned that the type II CD20 mAb obinutuzumab is particularly suited to eliminate brain B cells, with its efficient extravascular B cell depletion properties due to its superior peripheral and lymphoid B cell depletion potential.^31^, ^36^ Obinutuzumab not only mediates Fc effector‐dependent mechanisms but also induces Fc effector‐independent non‐apoptotic B cell death upon CD20 receptor binding.^28^, ^29^, ^31^

We have engineered a fusion between obinutuzumab and our Brainshuttle module (Brainshuttle‐CD20) to further enhance the potential clinical efficacy in the MS population by maximizing brain penetration. It is hypothesized that by directly targeting brain compartmentalized B cells in addition to peripheral B cells, Brainshuttle‐CD20 has the potential to improve patient response rates compared with current therapies that target the peripheral immune system. Moreover, the P329GLALA (PGLALA) mutation was introduced to silence Fc effector functions of the Brainshuttle‐CD20 mAb to improve safety while retaining B‐cell‐depleting properties.^37^, ^38^ Importantly, obinutuzumab containing the PGLALA mutation continues to deplete B cells in whole blood and mediates antitumour efficacy in B cell lymphoma xenograft models as opposed to the Fc‐silenced version of rituximab, which loses B cell depletion activity in absence of Fc effector functions.^37^

Anaemia has been identified as a potential safety concern associated with TfR1‐targeting mAb delivery technologies, particularly when Fc‐competent mAbs are used as cargos. This is attributed to the relatively high TfR1 expression on erythroblasts,^39^, ^40^ and the potential cross‐linking of effector cells via Fc‐binding with Fc‐competent antibodies. This may result in the killing of TfR1‐expressing cells, as well as other acute clinical signs related to downstream effects or Fc effector function; for instance, decreased circulating erythrocytes/reticulocytes,^41^ and acute infusion reactions.^39^, ^42^ We previously demonstrated in preclinical in vitro humanized murine models and in vivo transgenic mouse models that based on the Brainshuttle tertiary structure, the mAb Fc‐effector function of the cargo may be masked for binding to effector cells when bound to TfR1, but fully active when binding to the CNS target.^42^ The proof‐of‐concept studies for the Brainshuttle Alzheimer's disease mAb, trontinemab, further characterized receptor‐mediated transcytosis across the BBB in non‐human primates (NHPs), which was well tolerated at a dose of 10 mg/kg.^11^

Here, we aim to understand the potential risk of systemic exposure to Brainshuttle‐CD20 in blood and lymphoid organs. In particular, we investigate safety concerns related to Fc competence in combination with TfR1 targeting; Fc‐competent (wild‐type [WT]) and Fc‐silent (PGLALA) Brainshuttle‐CD20 mAbs are directly compared in their efficacy and safety in preclinical models. We assess the impact of adding a TfR1 binder and whether the inclusion of effector function affects in vitro and in vivo efficacy and safety of a type II CD20 binder, using dedicated models. We demonstrate that the Fc‐silenced Brainshuttle‐CD20 construct has a superior safety profile compared with the Fc‐competent construct while maintaining the ability to cross the BBB and deplete B cells. As a result, this is the first demonstration of effective B cell depletion in NHPs with an Fc effector‐silent CD20‐targeting antibody.

## 2 METHODS

### 2.1 Experimental design

**FIGURE 1:**
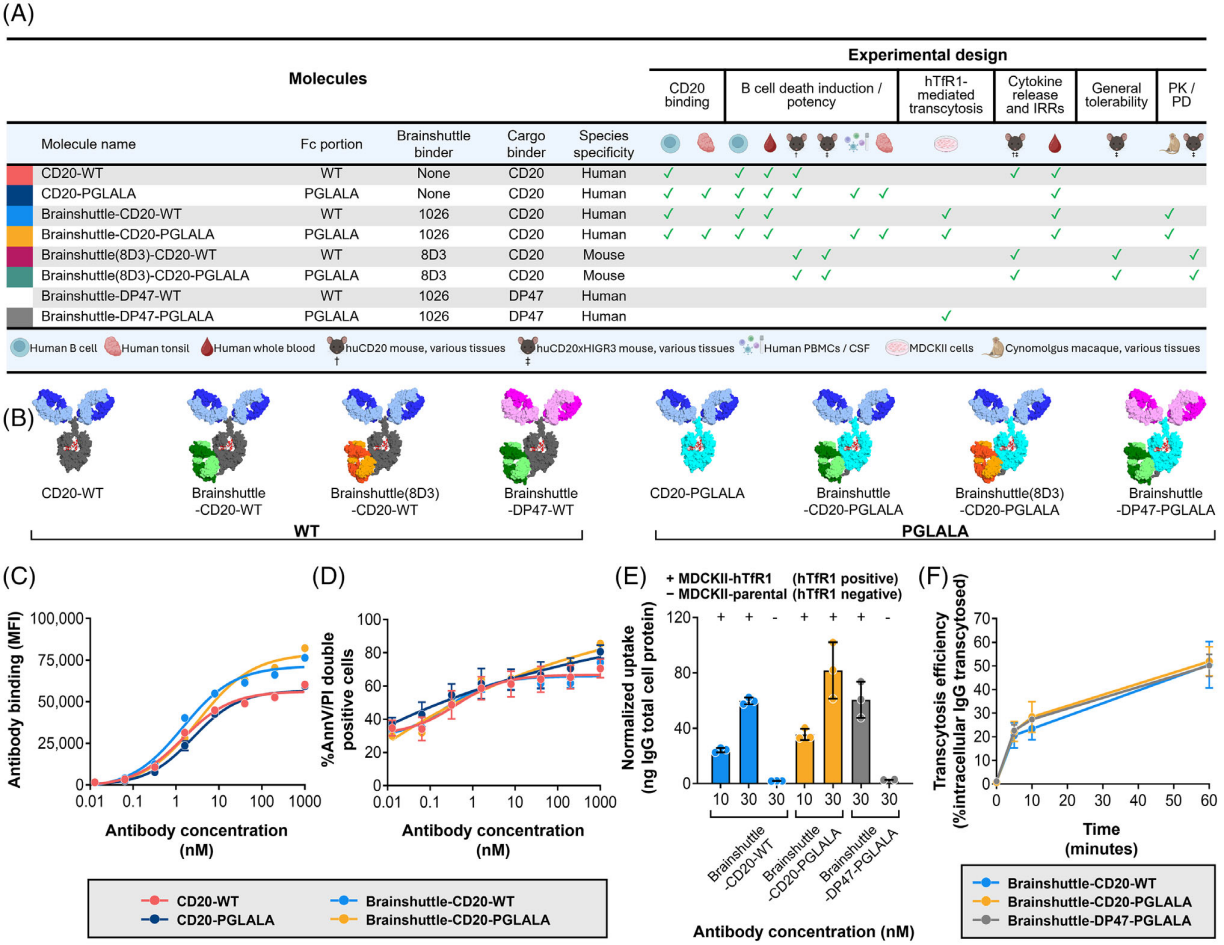
Binding, B cell killing, and uptake of Fc‐silent and Fc‐competent Brainshuttle CD20 mAbs. (A) Properties of the different Brainshuttle and control mAbs, with overview of experimental design and model systems used. In some cases, certain assays contributed to multiple categories of evaluation, reflecting their broader utility across different experimental criteria. (B) Schematic representations of Brainshuttle and control mAbs. Grey = Fc‐competent component of IgG1; turquoise = Fc‐silent component of IgG1; red = glycan; blue = anti‐human CD20 Fab arms of IgG1; magenta; non‐CD20‐binding Fab arms of IgG1; green = hTfR1 cross‐Fab; orange = murine‐TfR1 cross‐Fab. (C) FACS analysis of Z‐138 cell binding with Brainshuttle‐CD20 and control mAbs. (D) Direct B cell killing induced by binding of Brainshuttle‐CD20 and control mAbs to Z‐138 cells measured by annV assay and PI uptake. Results expressed as uptake of IgG normalized to total cell protein. (E) hTFR1‐mediated intracellular uptake of Brainshuttle‐CD20 and control mAbs into MDCKII‐hTfR1 and MDCKII‐parental cells (non‐hTfR1 expressing), normalized to total cell protein. No detectable uptake of either compound by MDCKII‐parental cells was observed, confirming that mAb uptake in hTfR1‐transfected cells was mediated by hTfR1. (F) Transcytosis efficiency derived from the mean amount of IgG transcytosed versus the mean amount of IgG in the corresponding intracellular compartment at the beginning (*T* = 0) of the chase (expressed as a percentage). Data represents the mean ± SD of triplicate measurements in (C–F). annV/PI, annexin V/propidium iodide; CSF, cerebrospinal fluid; FACS, fluorescence‐activated cell sorting; hTfR1, human transferrin receptor 1; huCD20, humanized CD20; huCD20xHIGR3, C57BL/6 huCD20xC57BL/6‐Tg(hIg‐γ1,κ,λ)ait mice; IgG, immunoglobulin G; MDCKII, Madin–Darby canine kidney II; MFI, mean fluorescence intensity; PBMCs, peripheral blood mononuclear cells; PGLALA, P329GLALA mutation (Fc‐silent); PK / PD, pharmacokinetic / pharmacodynamic; SD, standard deviation; WT, wild‐type (Fc‐competent).

To address potential safety concerns related to Fc competence or the Brainshuttle module, the safety and efficacy profiles of Brainshuttle and control mAbs were directly compared in preclinical models. CD20 binding and direct cell death induction were assessed in vitro in a human B cell lymphoma line (Z‐138; human mantle cell lymphoma), followed by a human whole blood assay (WBA) to assess B cell depletion and cytokine release. B cell depletion in ex vivo human tonsil and cerebrospinal fluid (CSF)/human peripheral blood mononuclear cell (PBMC) assays were also assessed. TfR1‐mediated cellular uptake and transcytosis were assessed in vitro to determine whether Fc silencing impacted TfR1‐mediated transcytosis of Brainshuttle‐CD20 mAbs. mAb toxicity profiles were compared in vivo in humanized BalbC or C57BL/6 CD20 (huCD20) and C57BL/6 huCD20xC57BL/6‐Tg(hIg‐γ1,κ,λ)ait (huCD20xHIGR3) transgenic mice, with assessment of clinical signs, cytokines, body temperature, clinical pathology, pharmacokinetics (PK)/pharmacodynamics (PD), and histopathology. Brainshuttle‐CD20 mAb toxicity was also assessed in cynomolgus macaques, with endpoints including clinical observations, body weight, body temperature, clinical pathology (haematology, coagulation, and clinical chemistry), immunophenotyping, cytokine evaluation, and histology (for the Fc‐silent Brainshuttle‐CD20 PGLALA mAb only). Each antibody construct used in the in vitro and in vivo models is summarized in Figure 1A.

### 2.2 Animal husbandry

All mouse studies were conducted at Pharma Research, Basel, which is fully accredited by the Association for Assessment and Accreditation of Laboratory Animal Care International (AAALACi) and were conducted with the approval of the Cantonal Veterinary Authority of Basel‐Stadt, following strict adherence to the Swiss federal regulations on animal protection. For all mouse studies, mice (up to six per cage [type IV macrolon boxes with autoclaved sawdust bedding]) were kept in a climate‐controlled animal room under periodic bacteriologic control, at 22 ± 2°C (monitored), at 40–80% humidity (monitored), with fluorescent lighting on a 12 h light/dark cycle, with background music that coordinated with light hours. The ventilation rate was more than 10 air exchanges per hour. Animals received food integrated into the cage lid, tap water ad libitum (in water bottles), and were offered an enriched environment. Prior to the study, mice were allowed to acclimatize to the test facility for at least seven days.

Cynomolgus macaques (*Macaca fascicularis*) studies were conducted at AAALACi‐accredited facilities in Germany (Study 1) and the UK (Study 2), in compliance with Directive 2010/63/EU (Study 1 and Study 2), the German Animal Welfare Act (Study 1) and the Animals (Scientific Procedures) Act 1986 amended in 2012 (Study 2). Animal procedures including humane endpoints were reviewed and approved by the applicable ethical review committee in each country. In Study 1, 12 naïve purpose‐bred females (22–26 months old) with a body weight ranging from 2.9 to 4.8 kg (at the start of dosing) were housed in pens in a climate‐controlled room that was maintained at 19–25°C and 40–70% relative humidity, with a minimum of eight air changes/hour, under an artificial 12 h light/dark cycle. Animals were paired and housed in stainless steel pens containing wood chips and environmental enrichment consisting of coloured plastic tools, coloured plastic balls, and stainless‐steel mirrors. They were fed with a certified diet (LabDiet 5048) twice daily, supplemented with fresh fruit and vegetables, and had access to ad libitum tap water. Animals were negative for tuberculosis (tested at the breeder) and bacterial disease (throat swab at pre‐dosing testing). Animals were acclimated for at least 2 weeks prior to dosing. In Study 2, eight purpose‐bred naïve females (between the ages of 1 year and 7 months and 3 years and 4 months old) with a body weight ranging from 2.3 to 3.6 kg (at the start of dosing) were housed in a climate‐control room that was maintained at 20–23°C and 29–68% relative humidity, with a minimum of 10 air changes/hour, and an under artificial 12 h light/dark cycle. Animals were housed in groups of 2–4 in pens containing wood shavings and environmental enrichment consisting of perches, plastic toys, balls, climbing frames, and stainless‐steel mirrors. They were fed with a certified diet (MPE Short SQC, Special Diet Services) once daily, along with a daily mix of fresh fruits, vegetables, forage mix, nuts, and biscuits, with access to ad libitum tap water. Animals were acclimated for 6 weeks prior to dosing.

### 2.3 Antibody and bispecific antibody production

Brainshuttle constructs were engineered by fusing a cross Fab (human; 1026) anti‐human TfR1 (hTfR1) antibody to the C‐terminus of a heavy chain from a human anti‐CD20 antibody (obinutuzumab; Figure 1A,B). Knobs‐into‐holes technology^43^ was used to favour the heterodimeric pairing of a heavy chain carrying the Brainshuttle module with a non‐fused heavy chain. CrossMAb technology^44^, ^45^ was used to avoid mispairing of the different light chains on the antibody complex. In the same way, the hTfR1 positive control reference shuttle, Brainshuttle‐DP47, was generated, except that the cargo binder was DP47 (a non‐target binding germline human immunoglobulin G [IgG] used as a blood tracer for brain contamination), instead of anti‐CD20. To generate the mouse TfR1 surrogate Brainshuttle, Brainshuttle(8D3), the single‐chain Fab (rat; 8D3) fragment of the murine anti‐TfR1 antibody was fused to the C‐terminus of the heavy chain from the human anti‐CD20 antibody, also utilizing knobs‐into‐holes technology. A glycine‐serine peptide linker was used to construct the single‐chain Fab (8D3) fragment. All Brainshuttle and Brainshuttle(8D3) mAb constructs were engineered in a WT Fc‐competent version of the anti‐CD20 antibodies and a version harbouring PGLALA Fc‐silencing mutation.^38^, ^44^ Further details on antibody production can be found in the Materials and Methods section of the Supporting Information.

### 2.4 CD20 binding

Z‐138 cells (a gift from the University of Leicester)^46^ were cultured as described in the Materials and Methods section of the Supporting Information. Cells were incubated with test antibodies and fluorescence‐activated cell sorting (FACS) analysis was performed to assess CD20 binding (see the Materials and Methods section of the Supporting Information for further details).

### 2.5 Cell death induction

Z‐138 cells were incubated with test antibodies (0.0128–1000 nM) for 24 h before measuring cell death by assessing annexin V (annV) expression and propidium iodide (PI) uptake as described within the Materials and Methods section of the Supporting Information.

### 2.6 TfR1‐mediated cell internalization and transcytosis

Parental Madin‐Darby canine kidney II (MDCKII) cells were cultured as described in the Materials and Methods section of the Supporting Information. MDCKII‐hTfR1 cells were generated by transient transfection with a DNA plasmid construct encoding hTfR1 (obtained from GenScript Biotech) using Lipofectamine 2000 (ThermoFisher Scientific [Invitrogen], 11668027); further transfection details are described in the Materials and Methods section of the Supporting Information. MDCKII cells were transiently transfected with hTfR1 24 h prior to transcytosis assays. hTfR1‐mediated internalization (uptake) and transcytosis (pulse‐chase) assays were performed 24 h post‐transfection; each treatment condition was evaluated in triplicate. Further details are described in the Materials and Methods section of the Supporting Information.

To measure intracellular IgG content, cells were lysed in RIPA Lysis and Extraction Buffer (ThermoFisher Scientific, 89900) containing protease inhibitors (Sigma Aldrich, 11697498001) and incubated for 30 min at 4°C (under frequent agitation). Solubilized cell lysates were transferred to 96‐well LoBind deepwell plates. Total cell protein content in cell lysates was determined using the Pierce BCA Protein assay (ThermoFisher, 23225), following the manufacturer's protocol. Samples from the chase phase were immediately diluted with assay buffer (1:1; see the Materials and Methods section of the Supporting Information) in 96‐well LoBind deepwell plates. All samples were stored at 4°C (or −80°C for longer periods) until ready for analysis. IgG content was quantitatively evaluated using a generic IgG enzyme‐linked immunosorbent assay (ELISA) with chemiluminescence‐based detection.

### 2.7 B cell subset depletion

B cells were transferred to 96‐well U bottom plates in a cultivation medium (1 × 10^6^ cells/well). Treatment antibodies were diluted and added at 1–100 nM and incubated for 8 h at 37°C, 5% CO_2_. Cells were then pelleted and processed for FACS staining. Cell viability was measured with the LIVE/DEAD and Zombie Aqua kits, the antibodies listed in Table S1, and BD Horizon Brilliant Stain Buffer (BD Biosciences, 563794). Samples were analyzed on a FACSymphony or FACS LSRFortessa using FACSDiva software (BD Biosciences). The phenotypic identification of B cell subsets was performed based on the surface marker expression (see the Materials and Methods section of the Supporting Information for further information); the staining strategy has been previously described.^47^

### 2.8 Human WBA

Fresh, undiluted human blood samples from six healthy individuals (after obtaining written consent; performed at the Roche Medical Center, Basel, Switzerland) were incubated for 24 h with concentrations of .01, .1, 1.0, 10, 100, 500, and 1000 nM of the Brainshuttle‐CD20 and control mAbs (Table S1). Subsequently, cytokine release (interleukin‐1 beta [IL‐1β], IL‐6, IL‐8, tumour necrosis factor‐alpha (TNF‐α), and interferon‐gamma [IFN‐γ]) and B cell depletion (only for the concentrations .01, 1.0, 100, and 1000 nM) were measured. Further information can be found in the Materials and Methods section of the Supporting Information.

### 2.9 Transgenic mice

In acute studies (potency and infusion‐related reaction [IRR] studies), the huCD20 model was used as no anti‐drug antibodies (ADAs) could arise in the short time frame of just a few hours. In longer‐term studies (immunization efficacy, single dose PK, and PK/PD studies) and the tolerability study, the huCD20xHIGR3 (B6‐Tg[MS4A1]Gne Tg[IGHG1, IGK, IGL]17Ait) mice were used to avoid impact of ADAs on the study results. For a key study (IRR study), both strains were used to allow the bridging of the two models. Both transgenic mouse models were from the C57BL/6 background, except for the potency study (wherein the huCD20 mouse was from a BalbC background). huCD20 mice were generated as the result of a random integration of a human CD20 bacterial artificial chromosome transgene as previously described.^48^, ^49^ C57BL/6‐Tg(hIg‐γ1,κ,λ)ait mice (HIGR3) were generated as previously described.^50^ Three transgenic constructs were used for co‐injection into pronuclei; these constructs contained unrearranged miniloci of the human Ig heavy chain γ1, the human Ig light chains κ, and λ, respectively. The transgenic mice inherited these transgenes at a single locus and subsequently expressed human IgG1 antibodies in their blood plasma.^50^ The huCD20xHIGR3 are the result of crossing the huCD20 and HIGR3 mouse lines; these mice are transgenic for the CD20 drug target and do not generate ADAs to human antibodies.

### 2.10 Potency study in huCD20 mice

Six female huCD20 mice per group, at the age of 8–16 weeks at the study start, were treated via intravenous (IV) administration with Brainshuttle(8D3)‐CD20 mAbs at .6, 1.3, and 13.3 mg/kg (∼210 kDa Brainshuttle[8D3]‐CD20‐WT, or Brainshuttle[8D3]‐CD20‐PGLALA), or .5, 1, and 10 mg/kg (equimolar concentrations) of parental (non‐shuttled) CD20 mAbs (∼150 kDa; CD20‐WT or CD20‐PGLALA) on study Days 0 and 3. On Days −1, 2, and 6, blood samples were taken for determination of frequencies of B cells (B220+ cells). On Day 6, mice treated with the Brainshuttle(8D3) compounds were sacrificed and the frequencies of B220+ B cells were determined in spleen and inguinal draining lymph node tissue. Spleens and inguinal lymph nodes from six naïve mice were harvested to set baseline B cell frequencies.

### 2.11 Immunization efficacy study in NP‐OVA immunized mice

huCD20xHIGR3 mice (*n* = 6 mice/group) were subcutaneously immunized with 20 µg of NP (4‐hydroxy‐3‐nitrophenylacetyl) coupled to ovalbumin (OVA), NP‐OVA (Biosearch Technologies, N‐5051)—a widely used T cell‐dependent antigen to induce NP‐specific B cell responses, including germinal centre (GC) formation—in alum on Day 0. On Days 5 and 8, mice were treated with IV 10 mg/kg of either Brainshuttle(8D3)‐CD20‐WT or Brainshuttle(8D3)‐CD20‐PGLALA. A vehicle control group was immunized with NP‐OVA but did not receive any B cell depletion agent. On Day 11, mice were sacrificed, and inguinal draining lymph nodes were harvested for determination of B cell depletion by flow cytometry as described below.

### 2.12 Flow cytometry immunization efficacy and potency studies

Absolute total B cell counts were determined using Trucount beads (BD Biosciences, 340335). Fc receptors were blocked with anti‐mouse CD16/CD32 antibodies (Biolegend 101320). Cells were sorted with an LSRFortessa cytometer (BD Biosciences) and analyzed using FlowJo software (BD Biosciences).

### 2.13 IRR and tolerability studies

A one‐day exploratory tolerability study was conducted in huCD20xHIGR3 mice to guide dose selection for further studies. Five groups of two male and two female mice/group were administered 0 (vehicle control), 3, or 10 mg/kg Brainshuttle(8D3)‐CD20‐PGLALA or Brainshuttle(8D3)‐CD20‐WT as a single IV bolus (5 mL/kg). Assessment of toxicity was based on mortality and clinical observations on the day of administration. Cytokine release and exposure assessment were evaluated in terminal retro‐orbital blood samples collected approximately 2 h post‐administration. IL‐2, IL‐6, IL‐10, monocyte chemoattractant protein‐1 (MCP‐1), macrophage inflammatory protein‐1β (MIP‐1β), macrophage inflammatory protein‐2 (MIP‐2), granulocyte colony‐stimulating factor (G‐CSF), IFN‐γ, TNF‐α, and keratinocyte‐derived cytokine (KC) serum cytokines were measured via Luminex assays (R&D Systems).

To determine the reason behind any differences in the maximum tolerated dose (MTD) toxicity profile and whether the effects were dependent on Fc activity and/or the Brainshuttle(8D3) module, Brainshuttle(8D3)‐CD20‐WT, Brainshuttle(8D3)‐CD20‐PGLALA, and non‐shuttled CD20‐WT were assessed in a single IV dose study in huCD20 and huCD20xHIGR3 transgenic mice. The acute phase response was characterized by assessing cytokine levels (IL‐2, MIP‐2, IL‐6, G‐CSF, IL‐10, IFN‐γ, MCP‐1, TNF‐α, MIP‐1β and KC, measured in serum 2 h from terminal retro‐orbital blood collection via Luminex assays) and body temperature (measured via telemetry; BMDS IPTT300 system [Plexx]) to identify the potential to generate IRRs. Cytokine release and exposure assessment were evaluated in terminal retro‐orbital blood samples collected approximately 2 h post‐administration.

### 2.14 PK and PD studies

Further details of each study listed below are available in the Materials and Methods section of the Supporting Information.

#### 2.14.1 Mouse single‐dose PK study in huCD20xHIGR3 mice

Brainshuttle(8D3)‐CD20 mAb PK was assessed after single‐dose IV bolus administration (13.3 mg/kg) in 12 female huCD20xHIGR3 mice. PK parameters were derived from composite concentration data and were estimated by non‐compartmental analysis using the kinetic evaluation program Phoenix@ Version 1.4.

At necropsy, one brain hemisphere was sampled and fixed in neutral buffered formalin for 24–48 h, trimmed, and embedded in paraffin blocks in coronal sections at six levels. Three sections per block were sliced at a thickness of 3 µm onto Superfrost Plus glass slides and immunohistochemistry was performed using the Ventana Discovery Ultra automated tissue stainer (Roche Tissue Diagnostics). Biotin conjugated donkey anti‐human IgG (Jackson ImmunoResearch, 709‐066‐149) was used to detect Brainshuttle(8D3) CD20 and the DP47 blood tracer using a horseradish peroxidase DAB Map detection kit (Ventana, 05266360001) and tissues were counterstained with Hematoxylin II (Ventana, 05277965001). Stained slides were digitized using a Hamamatsu NanoZoomer brightfield scanner at 40× magnification and digital slides were reviewed using the PMA Studio Software (Pathomation).

#### 2.14.2 PK/PD study in huCD20xHIGR3 mice

One control group (0 mg/kg) and six groups of 15 female huCD20xHIGR3 mice were treated with Brainshuttle(8D3)‐CD20‐WT or Brainshuttle(8D3)‐CD20‐PGLALA via single IV slow bolus administration (5 mL/kg). The control group was dosed with vehicle only. Exposure assessment, mortality, clinical observations, body weight, haematology, cytokine (IL‐6) level determination, inguinal lymph node and spleen organ weights, macroscopic findings, and histopathology of the inguinal lymph node, bone marrow, and spleen were subsequently evaluated. FACS analysis was performed in blood, spleen, and inguinal lymph nodes to assess the efficacy of both Brainshuttle(8D3)‐CD20 mAbs in depleting systemic and lymph node resident B cells (CD19+ B220+). Samples were analyzed by ELISA for the parent substances.

#### 2.14.3 Cynomolgus macaque single‐dose studies

Brainshuttle‐CD20‐WT and Brainshuttle‐CD20‐PGLALA were administered as a single IV bolus dose to female cynomolgus macaques in two separate studies, followed by a 15‐day observation period for Brainshuttle‐CD20‐PGLALA (10 mg/kg; Study 1) and an 8‐week observation period for Brainshuttle‐CD20‐WT (.1, 1.0, and 10 mg/kg; Study 2). In Study 1 and Study 2, four females were treated with each molecule at the 10 mg/kg dose. In Study 2 only, two females each were treated with the .1 and 1.0 mg/kg dose. For the purpose of comparison, only the 10 mg/kg dose groups from each study are described herein.

#### 2.14.4 Immunophenotyping and FACS cynomolgus macaque studies

FACS was performed to assess B cell depletion using flow cytometry and other lymphocyte immunophenotyping assessments. Further details are available in the Materials and Methods section of the Supporting Information.

### 2.15 Statistical analysis

For CD20 binding and direct B cell death induction, FACS gating was performed using FACSDiva Software (BD Biosciences) and the median fluorescence intensities as well as the percentage of positive cells were determined. Half maximal effective concentration (EC_50_) values were calculated based on a sigmoidal dose‐response (variable slope) analysis using GraphPad Prism (10.2.0). ANOVAs and two‐tailed paired t‐tests were also performed using GraphPad Prism. *p*‐values were considered significant if <.05. For quantifying blood B cell depletion in the potency and PK/PD study, applied generalized or linear mixed effect models were applied, which included the factors compound, concentration, and day as fixed effects (with full ternary interaction). The repeated‐measure structure of the data was described by a first‐order autocorrelation for a continuous time variable on the level of individual animals. Heteroscedasticity in the data was best fitted by allowing the model to use different variability estimates at each measurement day. All models were calculated in R, using packages nlme and emmeans. For IRR studies, temperature was measured in triplicate per animal and time point. Change in temperature was measured from pre‐dosing temperatures. For the mouse PK study, no formal statistical analysis was performed due to the composite study design. The data are summarized as arithmetic means only (or medians for time to peak drug concentration).

## 3 RESULTS

### 3.1 BBB transcytosis, retained CD20 binding affinity, and target‐mediated direct B cell killing in the presence of the Brainshuttle module and absence of Fc‐competence

The properties of all Brainshuttle and control mAbs are described in Figure 1A and mAb schematics are shown in Figure 1B. Throughout the experiments, anti‐human CD20 was used in combination with either human‐binding Brainshuttle or a mouse surrogate Brainshuttle(8D3), where a murine TfR1‐specific surrogate Fab fragment (8D3) was applied to mediate the shuttling function over the BBB for murine experiments. Brainshuttle alone refers to the human‐binding Brainshuttle module.

In CD20 binding assays, the Fc‐competent (Brainshuttle‐CD20‐WT) and Fc‐silent (Brainshuttle‐CD20‐PGLALA) mAbs demonstrated that the EC_50_ for binding was preserved, with a slightly higher maximal binding within the range of experimental variability (Figure 1C). These results indicate that Fc silencing and the Brainshuttle module do not impair CD20 binding. The extent of direct B cell death as a result of Brainshuttle‐CD20 mAb binding was comparable and concentration‐dependent for all mAbs tested (Figure 1D). Maximum B cell depletion in human CSF spiked with human PBMCs was similar for both Brainshuttle‐CD20‐PGLALA and non‐shuttled CD20‐PGLALA, and there were no significant differences between the two mAbs in terms of binding and B cell depletion in ex vivo human tonsil‐derived cells (Figures S1 and S2).

Brainshuttle‐CD20 mAbs were next evaluated for hTfR1‐mediated cellular uptake and transcytosis in vitro to address whether Fc silencing interferes with TfR1‐mediated transcytosis and to confirm that the CD20 constructs preserve the Brainshuttle module's transcytosis capabilities, independent of the Fc properties. Both Brainshuttle‐CD20 mAbs displayed 30‐fold higher intracellular uptake in MDCKII cells transfected with hTfR1 (MDCKII‐hTfR1) compared with hTfR1‐negative non‐transfected cells (MDCKII‐parental; Figure 1E). Following hTfR1‐mediated uptake, comparable transcytosis activity profiles were observed for both Brainshuttle‐CD20 mAb variants, with the amount of IgG released into the extracellular compartment being identical for both (Figure 1F). The hTfR1 positive control mAb, Brainshuttle‐DP47‐PGLALA—developed to investigate the potential influence of the shuttling target on efficacy and safety—showed similar hTfR1‐mediated uptake and transcytosis and, therefore, confirmed the functional activity of the hTfR1 binder when attached to the Fc region (WT or PGLALA) of the CD20 cargo (Figure 1F).

### 3.2 Potency study in huCD20 mice

**FIGURE 2:**
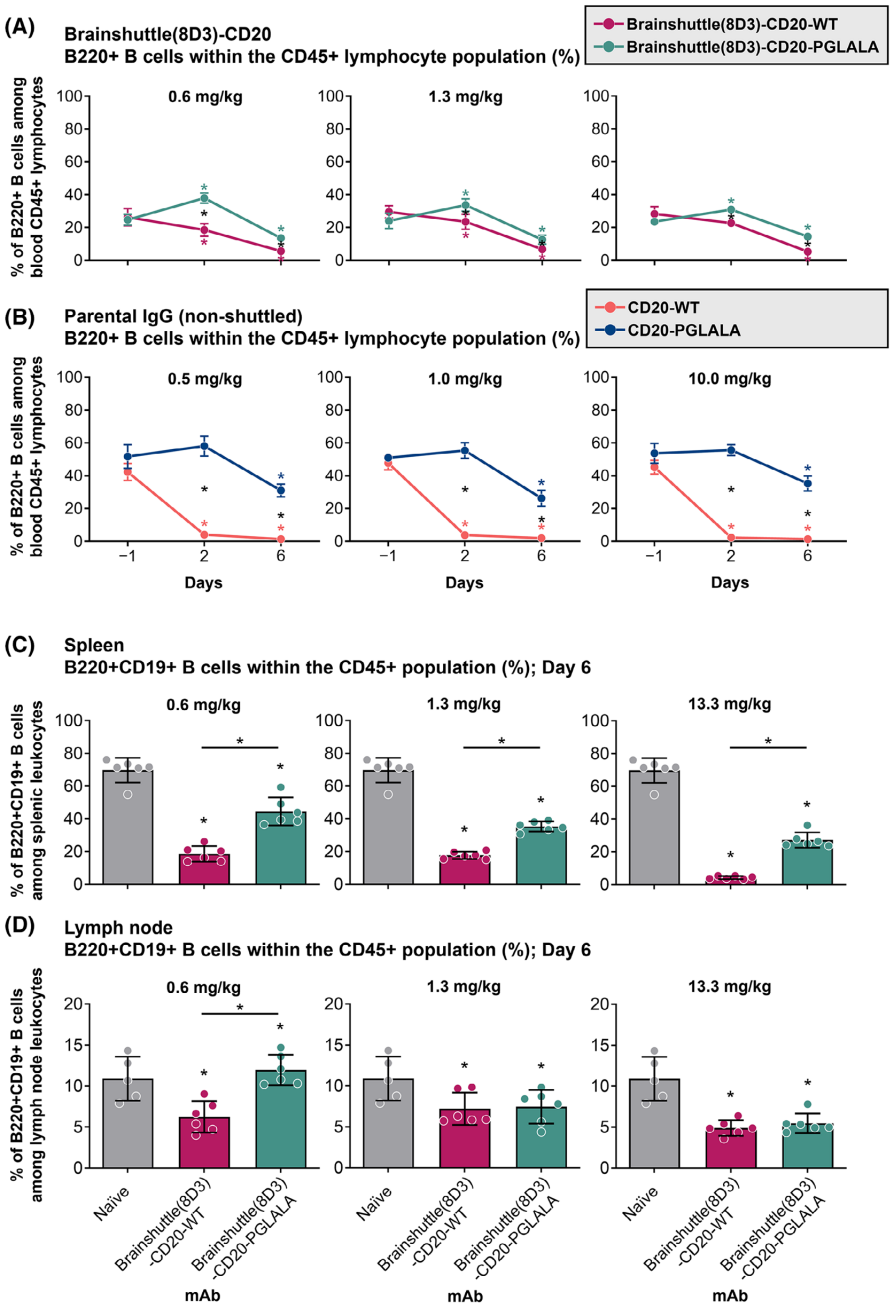
Kinetics of B cell depletion in blood and spleen. Kinetics of B cell depletion in huCD20 murine blood following administration of Brainshuttle(8D3)‐CD20 (A) and parental (non‐shuttled) (B) mAbs. B cell depletion is statistically significant from baseline with both molecules at later time points, and there is a significant difference between PGLALA and WT in both Brainshuttle(8D3) and parental molecules. Black asterisks represent statistically significant differences between molecules; coloured asterisks represent statistically significant differences to baseline. Kinetics of B cell depletion of Brainshuttle(8D3)‐CD20 mAbs at Day 6 in the spleen (C) and lymph node (D) of huCD20 and naïve mice. Only B cell numbers as a percentage of the CD45+ population are shown. Differences in the means of B cell depletion in the spleen between Brainshuttle(8D3)‐CD20‐WT and Brainshuttle(8D3)‐CD20‐PGLALA‐treated versus naïve mice were assessed by ANOVA. Significant differences in the treated groups versus naïve mice were seen at all dose groups. B cell depletion was significantly higher in Brainshuttle(8D3)‐CD20‐WT versus Brainshuttle(8D3)‐CD20‐PGLALA by two‐tailed paired *t*‐test at all dose groups. Data represents mean ± SD of *n* = 6 huCD20 mice. Data for the six naïve mice were used for comparison across all dose groups. ANOVA, one‐way analysis of variance; huCD20, humanized CD20; huCD20xHIGR3, C57BL/6 huCD20xC57BL/6‐Tg(hIg‐γ1,κ,λ)ait mice; mAb, monoclonal antibody; PGLALA, P329GLALA mutation (Fc‐silent); WT, wild‐type (Fc‐competent). *p‐values < .05.

To compare the impact of Fc‐silencing and the Brainshuttle module on B cell depletion, huCD20 transgenic mice were treated via IV administration with a surrogate mAb consisting of the CD20 cargo and a murine brainshuttle module (Brainshuttle[8D3]‐CD20) at .6, 1.3, and 13.3 mg/kg (Figure 2A), or .5, 1, and 10 mg/kg (equimolar concentrations) of parental (non‐shuttled) CD20 mAbs (Figure 2B). Blood B cell depletion, measured by the mouse pan‐B cell marker B220, was achieved with both Brainshuttle(8D3)‐CD20 mAbs on Day 6 (Figure 2A), without evidence of a dose‐dependent response, and there was a higher degree of B cell depletion with parental (non‐shuttled) CD20 mAbs compared with Brainshuttle(8D3)‐CD20 mAbs (Figure 2A,B). When comparing effects in lymphoid tissues, B cell killing by Brainshuttle(8D3)‐CD20‐WT was greater than by Brainshuttle(8D3)‐CD20‐PGLALA. The effect was more pronounced in the spleen (*p* < .05 at all doses) (Figure 2C) than in the lymph node (Figure 2D), where a difference between Fc‐silent and Fc‐competent Brainshuttle(8D3)‐CD20 mAbs was only observed in animals that received the lowest dose (*p* < .05 in .6 mg/kg dose group only; Figure 2D).

### 3.3 Immunization efficacy study in NP‐OVA immunized mice

**FIGURE 3:**
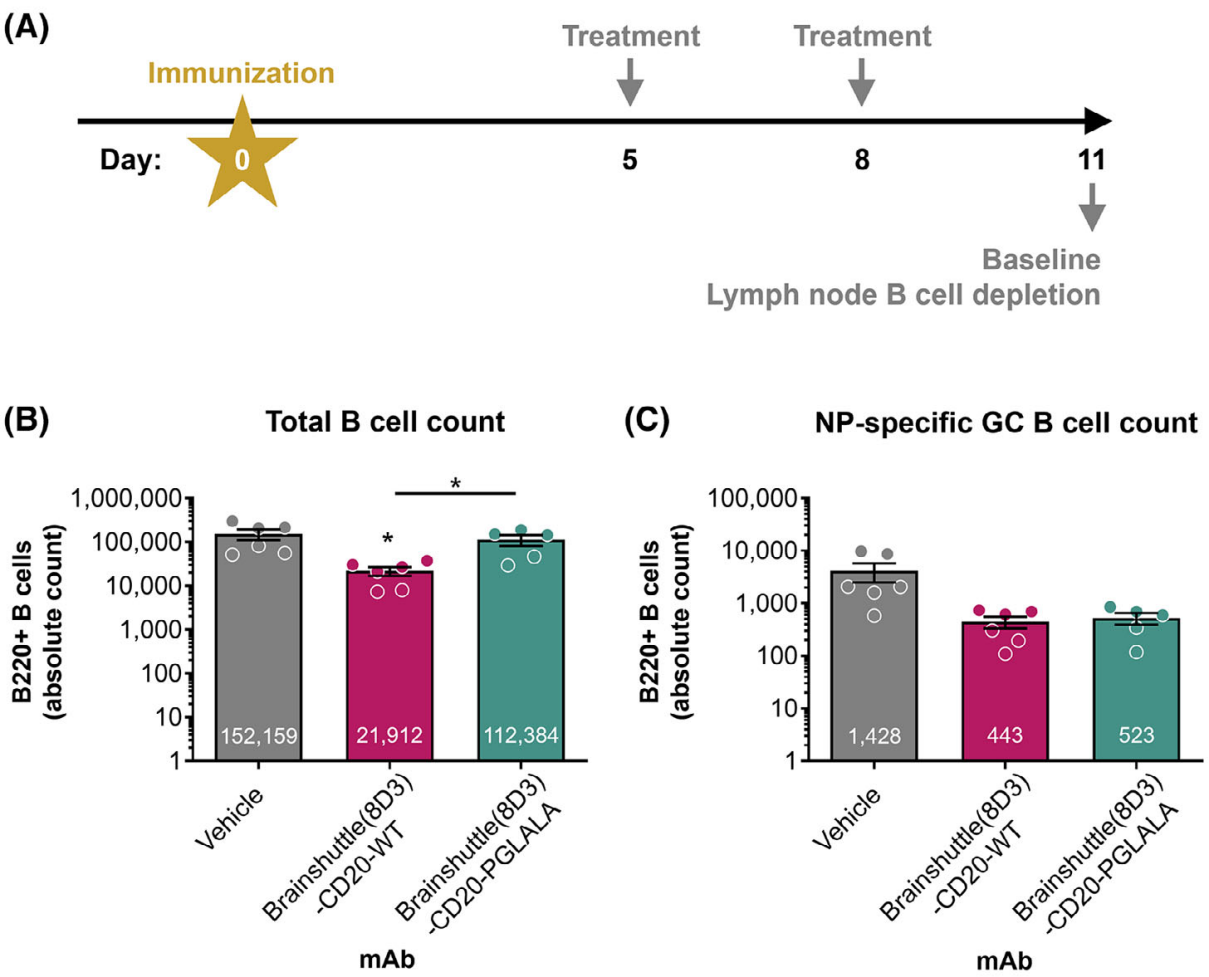
Depletion of B cell subpopulations in lymph nodes following NP‐OVA immunization and administration of Brainshuttle(8D3)‐CD20 mAbs. (A) Schematic representation of immunization and treatment of huCD20xHIGR3 mice. Depletion of either B220+ total lymph node B cells (B) or NP‐specific GC B cells (C) was assessed on Day 11 by flow cytometry. Depletion efficiency was determined by comparing B cell counts between either Brainshuttle(8D3)‐CD20‐WT or Brainshuttle(8D3)‐CD20‐PGLALA with the vehicle‐only group (immunized/vehicle treatment) and assessed by ANOVA. A *t*‐test determined statistical differences between Brainshuttle(8D3) mAbs. Data represents mean ± SEM B cell counts of *n* = 5 huCD20xHIGR3 mice per group. Mean data values are shown in each bar. ANOVA, one‐way analysis of variance; GC, germinal center; huCD20xHIGR3, C57BL/6 huCD20xC57BL/6‐Tg(hIg‐γ1,κ,λ)ait mice; mAb, monoclonal antibody; NP, 4‐hydroxy‐3‐nitrophenylacetyl; OVA, ovalbumin; PGLALA, P329GLALA mutation (Fc‐silent); SEM, standard error of the mean; WT, wild‐type (Fc‐competent). *p‐value < .05.

In previous experiments in huCD20 transgenic mice, significant ADA formation against administered human Brainshuttle‐CD20 mAbs was observed (data not shown). To avoid immunogenicity as a confounding factor, we used a double transgenic strain, huCD20xHIGR3; a mouse expressing a mini repertoire of human IgG1 mAbs.^50^ Mice were immunized subcutaneously with NP‐OVA on Day 0, followed by administration of 10 mg/kg IV doses of Brainshuttle(8D3)‐CD20 mAbs on Days 5 and 8 post‐immunization (Figure 3A). On Day 11 (peak of GC response to NP‐OVA), mice were sacrificed and the number of different B cell subsets were analyzed (Figure 3B). Only Brainshuttle(8D3)‐CD20‐WT led to substantial total B cell depletion (mainly IgD+ IgM+ naïve B cells; *p* < .05) compared with the vehicle‐only group (Figure 3B), while Brainshuttle(8D3)‐CD20‐PGLALA was efficient in depleting NP‐specific GC B cells, where the degree of depletion was similar to that achieved with Brainshuttle(8D3)‐CD20‐WT (Figure 3C).

### 3.4 Reduced risk of IRRs with Fc‐silent Brainshuttle‐CD20

A potential concern regarding Fc‐competent Brainshuttle mAbs is the induction of IRRs, which involves the cross‐linking of TfR1 on peripheral cells with Fc gamma receptors on immune cells and their subsequent activation. We previously reported that Brainshuttle(8D3)‐mAb fusion constructs with full effector function can be transported in “stealth mode” in the periphery due to steric hindrance while retaining full activity when engaged with their CNS target in preclinical in vitro and mouse models.^42^ IRR risk related to Fc effector function and the presence of the Brainshuttle module was explored in an in vitro human WBA, as well as in huCD20 and huCD20xHIGR3 mice by measuring cytokine release in vitro and temperature change in vivo.

**FIGURE 4:**
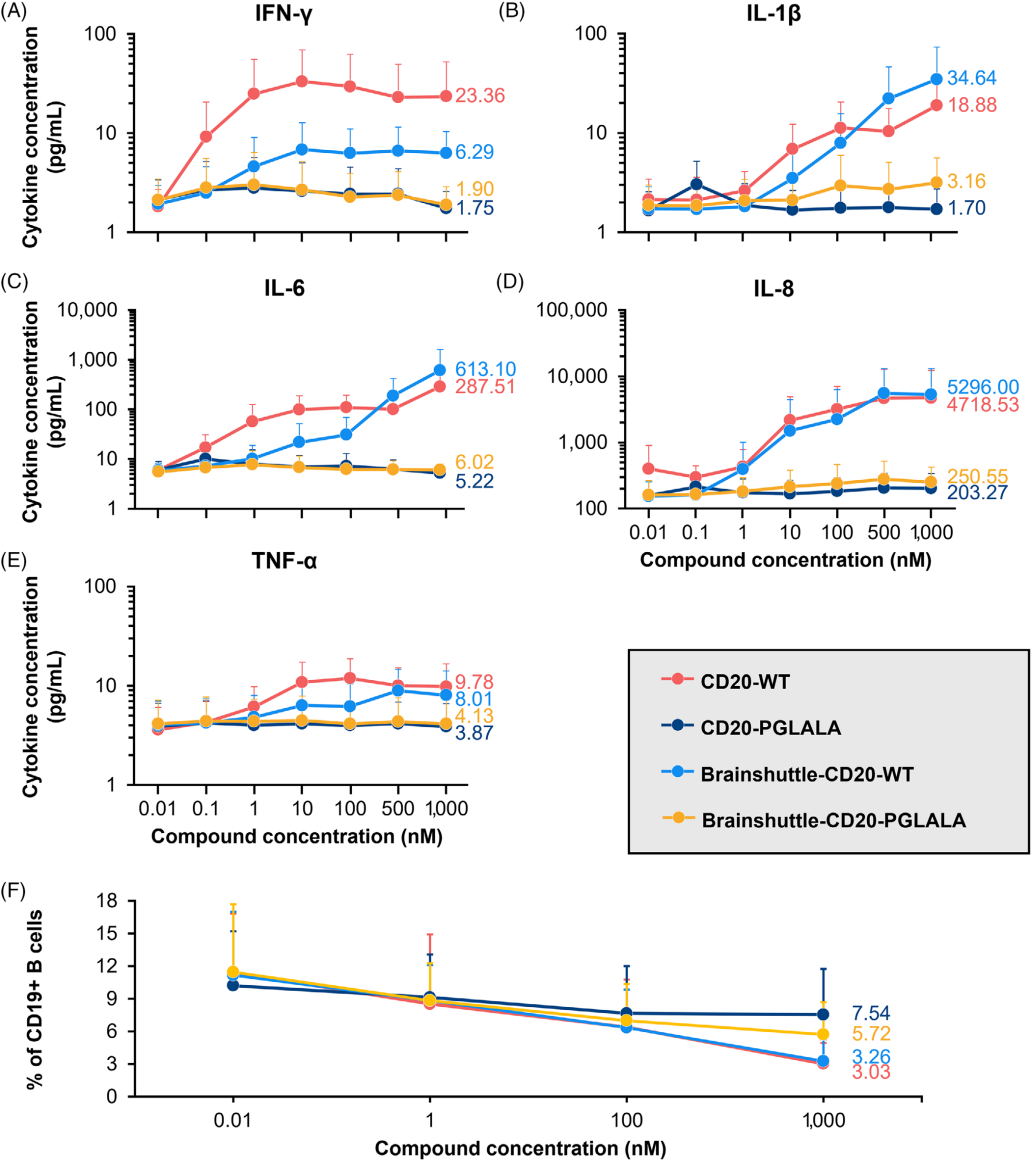
Cytokine release is associated with Fc‐competence. Cytokine concentrations in human WBAs after 24 h incubation of healthy human blood with non‐shuttled and shuttled mAbs (A–E). (F) Flow cytometry of CD19+ B cells in human WBAs. Data represents the mean +SD of *n* = 6 healthy blood donors. Mean data values for the 1000 nM compound concentrations are displayed. IFN‐γ, interferon‐gamma; IL‐1β, interleukin‐1 beta; IL‐6, interleukin‐6; IL‐8, interleukin‐8; PGLALA, P329GLALA mutation (Fc‐silent); SD, standard deviation; TNF‐α, tumour necrosis factor‐alpha; WBAs, whole blood assays; WT, wild‐type (Fc‐competent).

In a human WBA, no noteworthy release of any investigated cytokine was observed for CD20‐PGLALA and Brainshuttle‐CD20‐PGLALA within the concentration range examined (.01–1000 nM; Figure 4A–E). In contrast, moderate IFN‐γ, TNF‐α, IL‐6, and IL‐1β, and strong IL‐8 cytokine release were observed for CD20‐WT (Figure 4A–E). Moderate‐to‐strong IL‐6 and strong IL‐8 cytokine release were observed for Brainshuttle‐CD20‐WT at the highest concentration (Figure 4C,D). All tested mAbs demonstrated concentration‐dependent B cell depletion; however, Fc‐silent PGLALA variants were less potent compared with the corresponding Fc‐competent WT variants (Figure 4F). The comparable Brainshuttle‐CD20‐WT and CD20‐WT B cell depletion potency in the WBA indicates that the Brainshuttle module has no detrimental impact on function. These data indicate that while type II anti‐CD20 compounds harbouring WT Fc‐competent effector functions have a higher efficacy for B cell depletion, they also display a higher propensity for cytokine release compared with Fc‐silent PGLALA variants.

A tolerability study was conducted in huCD20 and huCD20xHIGR3 mice to determine the MTD of Brainshuttle(8D3)‐CD20 mAbs after a single IV injection. Treatment with Brainshuttle(8D3)‐CD20‐WT and Brainshuttle(8D3)‐CD20‐PGLALA at 3 mg/kg and 10 mg/kg (*n* = 2 animals/sex/dose group for each compound and mouse model) was well tolerated until scheduled euthanasia/sacrifice at 2 h post‐injection. No clinical signs were observed with the 3 mg/kg dose of Brainshuttle(8D3)‐CD20‐WT in either sex or in females at the 10 mg/kg dose; a hunched posture was recorded in both male mice treated with Brainshuttle(8D3)‐CD20‐WT at 10 mg/kg and one male was slightly hypoactive. No clinical signs were observed with Brainshuttle(8D3)‐CD20‐PGLALA treatment at either dose. There was no difference in findings between hCD20 and hCD20xHIGR3 mouse models.

**FIGURE 5:**
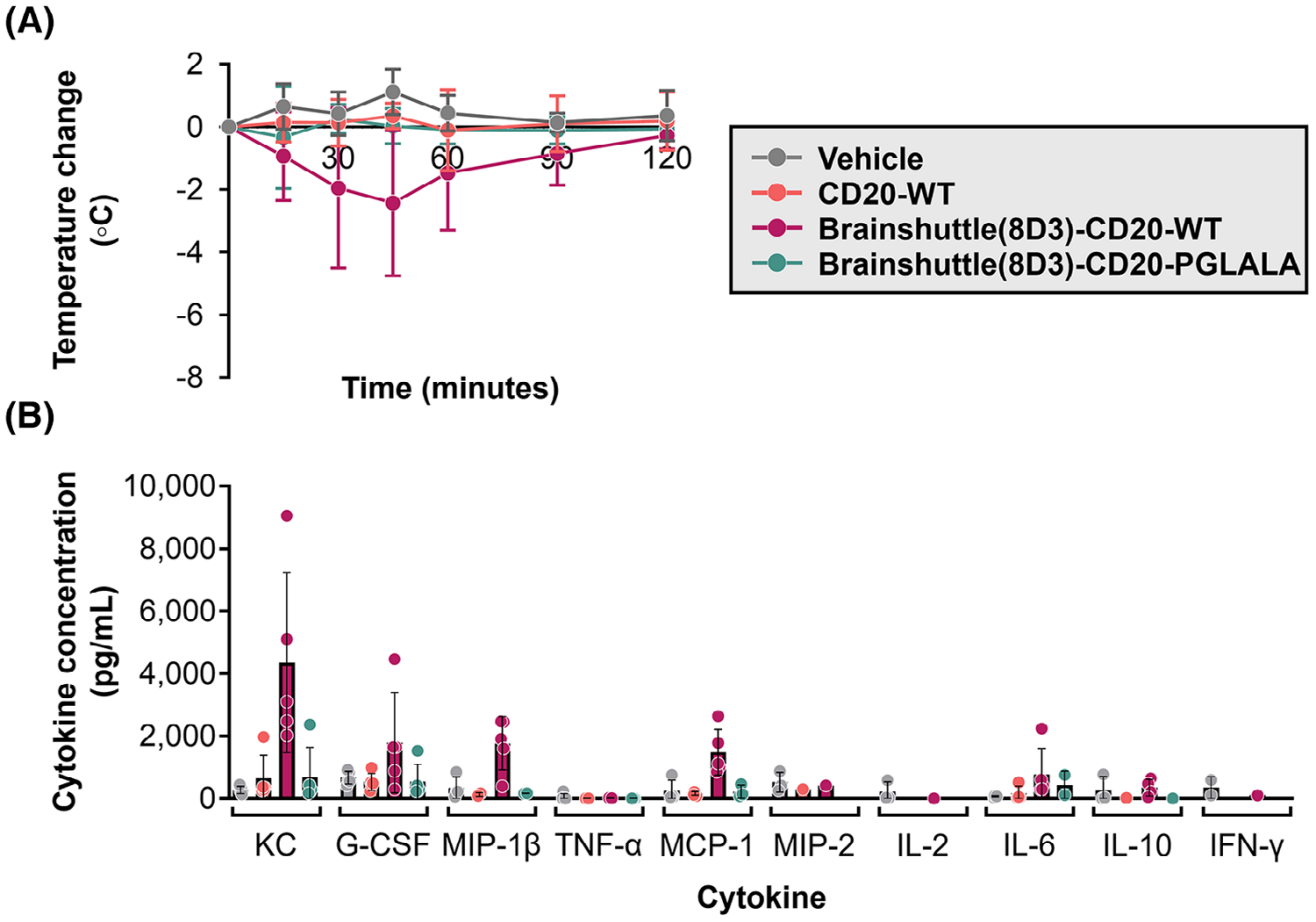
Effect of Fc silencing on body temperature and cytokine levels in huCD20xHIGR3 mice. (A) Effects of Brainshuttle(8D3)‐CD20 mAbs on body temperature in huCD20xHIGR3 transgenic mice following IV administration at 10 mg/kg compared with non‐shuttled CD20‐WT and administration of vehicle only. (B) Cytokine levels measured 2 h after administration of mAbs. Data points represent mean ± SD of *n* = 5 huCD20xHIGR3 mice per group. Corresponding data for huCD20 mice is shown in Figure S3. G‐CSF, granulocyte colony‐stimulating factor; huCD20, humanized CD20; huCD20xHIGR3, C57BL/6 huCD20xC57BL/6‐Tg(hIg‐γ1,κ,λ)ait mice; IFN‐γ, interferon‐gamma; IL, interleukin; IV, intravenous; KC, keratinocyte‐derived cytokine; mAbs, monoclonal antibodies; MCP, monocyte chemoattractant protein; MIP, macrophage inflammatory protein; PGLALA, P329GLALA mutation (Fc‐silent); SD, standard deviation; TNF‐α, tumour necrosis factor‐alpha; WT, wild‐type (Fc‐competent).

To determine the reason behind these differences in toxicity profiles and whether the effects were dependent on Fc activity and/or the Brainshuttle(8D3) module, Brainshuttle(8D3) mAbs, and non‐shuttled CD20‐WT were assessed in a subsequent single dose study in both huCD20 and huCD20xHIGR3 mice (*n* = 5 animals/group, males/females mixed for each compound and mouse model). Slight‐to‐moderate hypoactivity was recorded 15 min after IV administration of 10 mg/kg Brainshuttle(8D3)‐CD20‐WT in all huCD20 and huCD20xHIGR3 mice. However, within 2 h after administration, all animals recovered and exhibited normal behaviour. Body‐temperature data demonstrate a clear differentiation between treatments; all animals treated with Brainshuttle(8D3)‐CD20‐WT experienced an acute temperature drop of approximately 2–5°C, indicative of an acute IRR, followed by a quick recovery, while animals treated with CD20‐WT or Brainshuttle(8D3)‐hCD20‐PGLALA experienced no drop in body temperature (Figure 5A; Figure S3A). Compared with Brainshuttle(8D3)‐CD20‐PGLALA, cytokine levels in Brainshuttle(8D3)‐CD20‐WT treatment groups indicated a typical pattern for classical IRR^51^ notably, KC, MCP‐1, G‐CSF, and MIP‐1β serum levels were elevated, with moderate IL‐6 release (Figure 5B; Figure S3B). Other cytokines remained largely unaffected, with values for TNF‐α, IL‐2, and IFN‐γ below the limit of detection (2.3, 1.0, and 1.1 pg/mL, respectively). In mice treated with Brainshuttle(8D3)‐CD20‐PGLALA, cytokine levels for KC, G‐CSF, MIP‐1β, MCP‐1, and MIP‐2 remained largely unaffected or declined moderately. Under study conditions, the MTD after a single IV administration was ≥10 mg/kg for both Brainshuttle(8D3)‐CD20 mAbs.

### 3.5 Fc‐silencing mitigates effects on reticulocytes in transgenic mice while maintaining blood B cell depletion properties

To determine their potential toxicity and toxicokinetic profile, Brainshuttle(8D3)‐CD20 mAbs were administered as a single IV dose (.6, 1.3, or 13.3 mg/kg) to female huCD20xHIGR3 mice. Following IV administration, all mice survived until scheduled sacrifice on Day 3, 8, or 22 (*n* = 5 each) post‐administration. No clinical signs or effects on body weight were observed. IL‐6 levels were below the lower limit of quantification (1.94 pg/mL) in all animals at all time points. Compared with vehicle control, animals treated with Brainshuttle(8D3)‐CD20‐WT (13.3 mg/kg) demonstrated severe decreases in absolute reticulocyte counts on Day 3 (−95%, *p* < .001), mainly due to a decrease in immature reticulocyte fraction, followed by an increase in reticulocyte counts on Day 8 (+40%, *p* < .01). Slightly decreased absolute lymphocyte counts (−53%, *p* < .01) on Day 8 in animals treated with Brainshuttle(8D3)‐CD20‐WT (13.3 mg/kg) was consistent with expected pharmacology of B cell killing. In contrast, there were no relevant haematological changes in animals treated with Brainshuttle(8D3)‐CD20‐PGLALA (data not shown).

**FIGURE 6:**
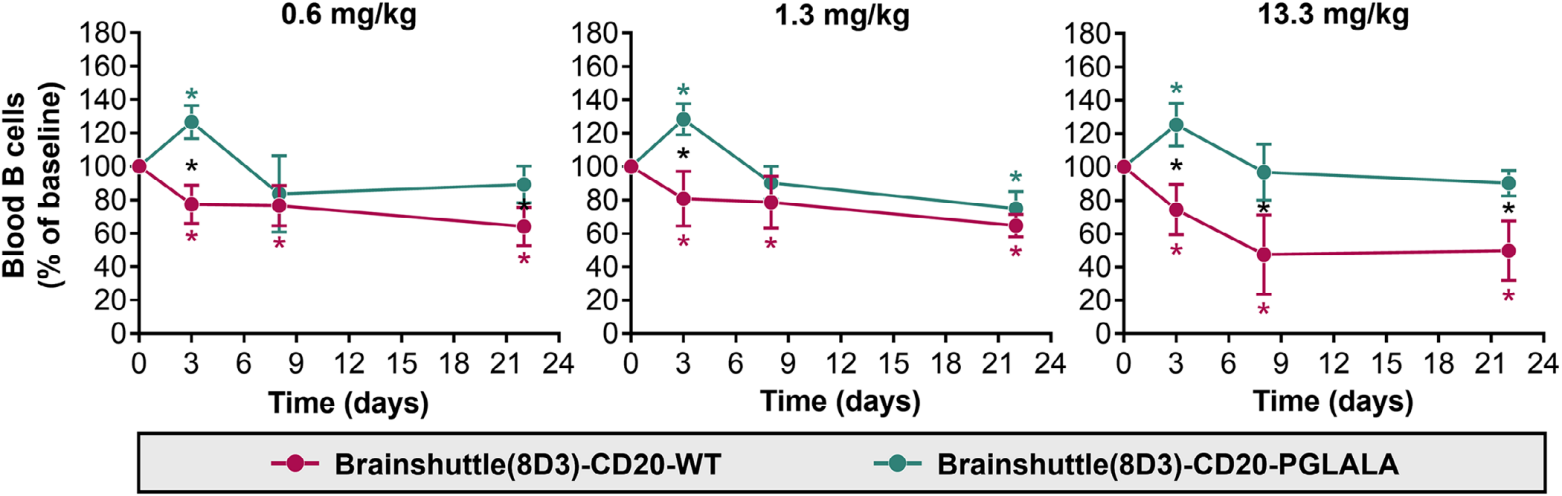
B cell depletion in response to treatment with Brainshuttle(8D3)‐CD20 mAbs by FACS analysis. The frequency of remaining B220+ B cells in blood was calculated by setting the percentage of B220+ cells at the baseline measurement to 100%. Data represents mean ± SD (at baseline and Day 3 *n* = 15, Day 8 *n* = 10, Day 22 *n* = 5 huCD20xHIGR3 mice per group [the reduced number of mice at each time point was due to the scheduled sacrifice of *n* = 5 mice per time point]). FACS, fluorescence‐activated cell sorting; huCD20xHIGR3, C57BL/6 huCD20xC57BL/6‐Tg(hIg‐γ1,κ,λ)ait mice; mAbs, monoclonal antibodies; PGLALA, P329GLALA mutation (Fc‐silent); SD, standard deviation; WT, wild‐type (Fc‐competent). *p‐values < .05. Black asterisks represent statistically significant differences between molecules; coloured asterisks represent statistically significant differences to baseline.

In blood, Brainshuttle(8D3)‐CD20‐PGLALA increased B cell numbers on Day 3 followed by depletion from Day 8 up to Day 22 at all three doses (Figure 6). Treatment with Brainshuttle(8D3)‐CD20‐WT led to ∼20% B cell depletion on Day 3, reaching a maximum of ∼50% depletion on Day 22 at the 13.3 mg/kg dose (Figure 6). On Day 8, treatment at .6 and 1.3 mg/kg led to a similar magnitude of B cell depletion (10–20%), whereas a larger depletion effect of Brainshuttle(8D3)‐CD20‐WT was observed at 13.3 mg/kg (Figure 6). In inguinal lymph nodes, the most pronounced reduction of B cell numbers (∼50%) was observed on Day 3 (Figure S4A). In the spleen, no effect of Brainshuttle(8D3)‐CD20‐PGLALA was observed (Figure S4B). There was no difference in the extent of hemosiderosis (evidence of iron accumulation) in the spleen between treated and control animals (Figure S5). There were also no histological findings in inguinal lymph nodes or bone marrow.

### 3.6 PK exposure and brain penetration in huCD20xHIGR3 transgenic mice

**FIGURE 7:**
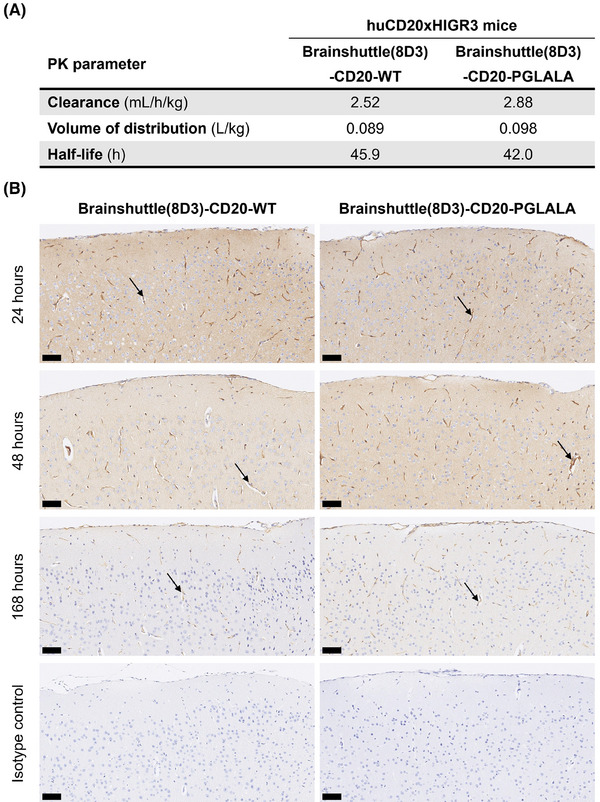
Comparable penetration of Brainshuttle(8D3)‐CD20 mAbs in the mouse brain. (A) PK parameters of Brainshuttle(8D3)‐CD20 mAbs in huCD20xHIGR3 mouse serum following IV administration at 13.3 mg/kg, derived from mean concentration versus time data for *n* = 12 huCD20xHIGR3 mice. (B) Human IgG immunohistochemical (brown) staining of the cerebral cortex in the brain of huCD20xHIGR3 mice treated with Brainshuttle(8D3)‐CD20 mAbs over 24, 48, and 168 h. Arrows indicate endothelial staining. Blue = hematoxylin counterstain. Scale bars = 50 µm. huCD20xHIGR3, C57BL/6 huCD20xC57BL/6‐Tg(hIg‐γ1,κ,λ)ait mice; IgG, immunoglobulin G; IV, intravenous; mAbs, monoclonal antibodies; PGLALA, P329GLALA mutation (Fc‐silent); PK, pharmacokinetic; WT, wild‐type (Fc‐competent).

Following single‐dose IV administration of 13.3 mg/kg Brainshuttle(8D3)‐CD20 mAbs, PK parameters in serum were calculated. PK parameters were similar between PGLALA and WT constructs (Figure 7A). Following injection of the DP47 blood tracer and perfusion to control for antibodies remaining within the brain vasculature, both Brainshuttle(8D3)‐CD20 mAbs were detected in brain tissue at 24, 48, and 168 h post‐administration, except for in one animal treated with Brainshuttle(8D3)‐CD20‐WT at 168 h, where the brain concentration was below the limit of quantification. Brain‐to‐serum ratios ranged between .0183 and .131 for Brainshuttle(8D3)‐CD20‐PGLALA and between .00951 and .153 for Brainshuttle(8D3)‐CD20‐WT (see the corresponding Results section and Figure S6 in the Supporting Information for further details).

Immunohistochemistry for human IgG, which detects the DP47 blood tracer and both Brainshuttle(8D3)‐CD20 mAbs, demonstrated differences in parenchymal staining intensity at each time point (Figure 7B), which was consistent with the brain PK profiles. For both Brainshuttle(8D3)‐CD20 mAbs, the strongest diffuse brain parenchymal and meningeal vessel human IgG staining was at 24 h post‐administration, with mostly only vascular endothelial cell staining at the 168 h time point, consistent with the presence of the blood tracer (Figure 7B).

### 3.7 Fc‐silenced Brainshuttle‐CD20 depletes B cells in cynomolgus macaques with a favourable safety profile

The cynomolgus macaque is the only cross‐reactive species for both the CD20‐ and TfR1‐binding moieties of the Brainshuttle‐CD20 mAb clinical candidate. While the mouse models are useful to generate the initial proof‐of‐concept and understanding, the cynomolgus macaque model is more suitable for making assessments regarding B cell depletion. PK/PD and tolerability studies in cynomolgus macaques were performed to assess the PK/PD and safety profile of Brainshuttle‐CD20 mAbs following single‐dose IV administration as part of the drug candidate selection for clinical development. Two separate studies were conducted, from which four animals were compared in the 10 mg/kg dose group. Animals were treated with a single IV slow bolus of 10 mg/kg Brainshuttle‐CD20‐PGLALA with a 15‐day observation period followed by euthanasia and necropsy (Study 1), or .1, 1.0, or 10 mg/kg Brainshuttle‐CD20‐WT with an 8‐week observation period (Study 2). Evaluated in‐life parameters included clinical observations, body weight, body temperature, clinical pathology (haematology, clinical chemistry, and coagulation), immunophenotyping, and cytokine evaluation. For Brainshuttle‐CD20‐PGLALA, organ weights, macroscopic examination, and histological examination were also performed. For the purposes of comparison, only the 10 mg/kg doses of Brainshuttle‐CD20‐WT and Brainshuttle‐CD20‐PGLALA were compared.

**FIGURE 8:**
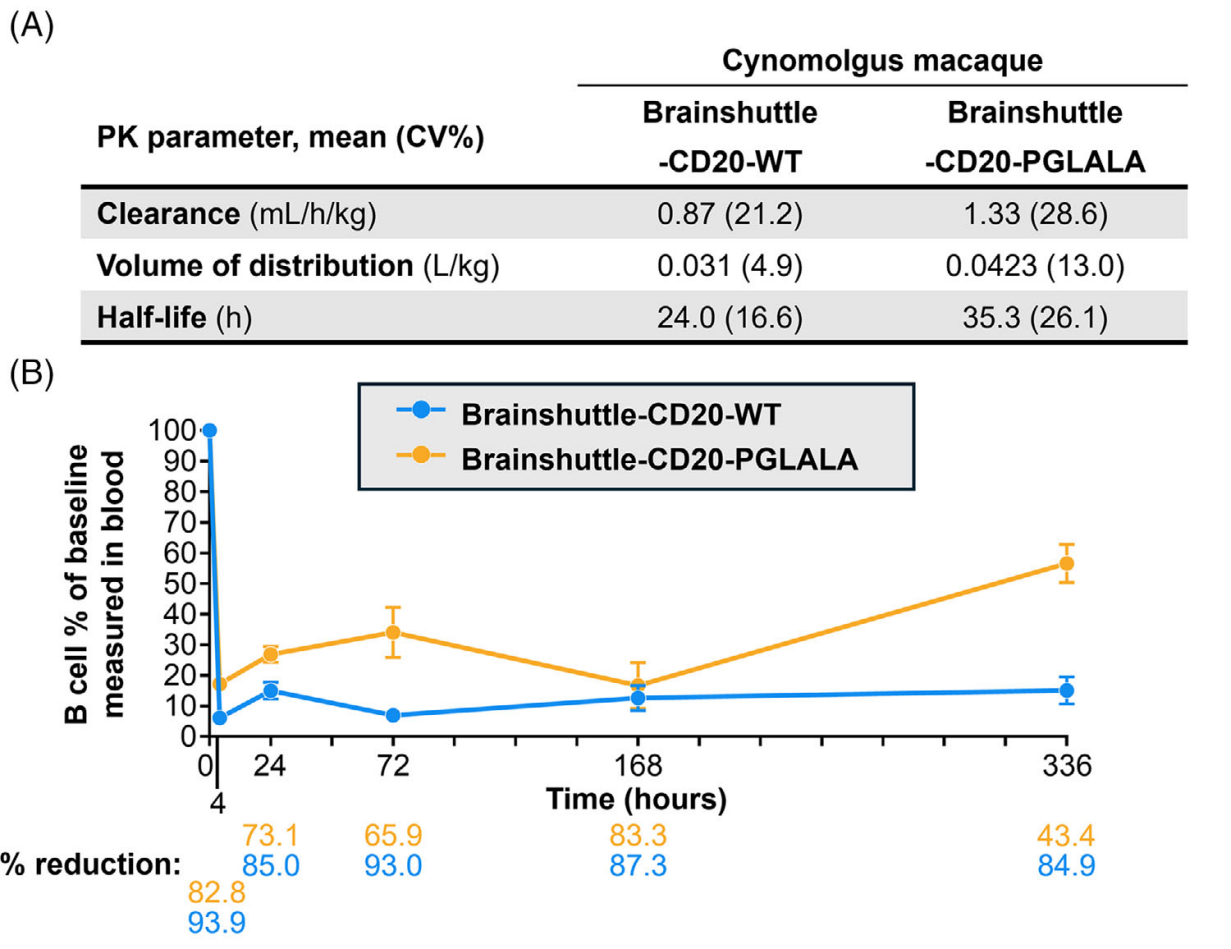
B cell depletion following Brainshuttle‐CD20 administration in cynomolgus macaques. (A) PK parameters of both Brainshuttle‐CD20 mAbs from two independent PK studies. (B) B cell numbers following Brainshuttle‐CD20 mAb administration are presented as a percentage of B cells measured at baseline. Data represents mean ± SD of *n* = 4 animals. mAbs, monoclonal antibodies; PGLALA, P329GLALA mutation (Fc‐silent); PK, pharmacokinetic; SD, standard deviation; WT, wild‐type (Fc‐competent).

Following the administration of Brainshuttle‐CD20 mAbs, PK parameters in cynomolgus macaque serum were similar (Figure 8A). In Study 1, the CSF concentration of Brainshuttle‐CD20‐PGLALA showed good brain penetration, with an area under the concentration‐time curve (AUC) from 0 to 168 h of 47.2 ng/h/mL based on the composite profile. CSF concentrations measured in individual samples of NHPs following a single IV bolus of 10 mg/kg of Brainshuttle‐CD20‐PGLALA ranged from .183 to 4.71 nmol/L in the 24–168 h time window (composite concentrations are shown in Figure S7A). Plasma concentrations are shown in Figure S7B, and the AUC for the 0–168 h time window was 39700 nmol*h/L, with a CSF‐to‐Plasma ratio of .6%. Brainshuttle‐CD20‐PGLALA was well tolerated in all animals and there were no treatment‐related changes in clinical observations, body weight, body weight development, or body temperature. There were also no safety‐relevant effects on blood cytokine levels, clinical chemistry (including transferrin, iron, ferritin, unsaturated iron binding capacity, and total iron binding capacity), and macroscopic or histological features. Minimal‐to‐mild and transient clinical pathology changes in animals treated with Brainshuttle‐CD20‐PGLALA were limited to slight decreases in mature red cell mass parameters (red blood cell [RBC] count, haemoglobin, and hematocrit) and increases in reticulocyte counts, which were attributed to the multiple blood collections. In addition, there was a transient increase in the absolute neutrophil count in all animals at 4 h post‐dose on Day 1, which recovered thereafter. There was also a transient increase in IL‐6 levels at the 1‐ and 4 h time points.

In study 2, Brainshuttle‐CD20‐WT was generally well tolerated, except in one 10 mg/kg‐treated animal, which exhibited transient clinical signs of subdued and/or decreased activity on Days 2–3 post‐dosing. Overall, although there were some post‐treatment changes in cytokine levels in some animals, none appeared to be proportional to the administered dose. As expected, Brainshuttle‐CD20‐WT administration at 10 mg/kg induced transient changes in haematology, coagulation, and clinical chemistry parameters consistent with repeated blood collection and additionally, an acute phase response. Findings consistent with repeated blood collection included minimal decreases in mature RBC mass parameters on Day 8 and increases in absolute reticulocyte counts. Findings consistent with acute phase response included increases in fibrinogen, C‐reactive protein, haptoglobin, and ferritin concentrations, as well as minimal decreased iron concentration on Days 2 and 8.

The expected PD effect of lower lymphocyte numbers for both constructs was observed microscopically in lymphoid organs, including mesenteric lymph nodes and spleen (PGLALA), along with decreased peripheral blood CD19+ B cells, and CD86‐activated B cell counts starting at 4 h after dosing until Day 15 (Brainshuttle‐CD20‐PGLALA [Study 1]) or Day 41 (Brainshuttle‐CD20‐WT [Study 2]). In both cynomolgus macaque studies, there was a rapid decrease in blood B cell numbers following administration of both mAbs. The maximum mean reduction in B cell count at 4 h post‐dosing was 94% for Brainshuttle‐CD20‐WT and 83% for Brainshuttle‐CD20‐PGLALA (Figure 8B). A maximum decrease in T cell count was observed with both mAbs at 4 h post‐dosing (80% reduction with Brainshuttle‐CD20‐WT, 25% reduction with Brainshuttle‐CD20‐PGLALA); this decrease was transient, with a return to baseline levels after 24–96 h. The maximum decrease for natural killer cells was also observed 4 h post‐dosing for both mAbs (83% reduction with Brainshuttle‐CD20‐WT, 50% reduction with Brainshuttle‐CD20‐PGLALA); this decrease was also transient, with a return to baseline levels after 96–168 h. These observed effects were likely due to transient extravasation and subsequent recovery. There was a transient 100% increase observed for monocyte levels 4 h post‐dosing which reached baseline levels after 24 h with Brainshuttle‐CD20‐WT and no change with Brainshuttle‐CD20‐PGLALA. The transient increase of monocytes is potentially due to Fc effector function mediated transient activation of monocytes. All animals developed ADAs, independent of the Fc variant status of the Brainshuttle‐hCD20 mAbs. ADAs in NHPs are considered to not be predictive of the nature of immunogenicity in humans, and obinutuzumab is known to be immunogenic in NHPs.^52^, ^53^

## 4 DISCUSSION

The ability to achieve sufficient brain penetration while maintaining a safe therapeutic window is key for brain‐targeted antibody development. To this end, we engineered an Fc‐silent Brainshuttle‐CD20 mAb as a bispecific modular fusion protein of a TfR1‐directed brain shuttle and the obinutuzumab anti‐CD20 mAb with the PGLALA mutation to abolish Fc‐mediated immune effector functions. In a head‐to‐head comparison in human and mouse in vitro models, and in in vivo mouse and cynomolgus macaque preclinical models, the Fc‐silenced Brainshuttle‐CD20‐PGLALA mAb demonstrated a superior safety profile compared with an Fc‐competent construct (Brainshuttle‐CD20‐WT) while maintaining the ability to achieve higher brain exposures compared with a “normal” mAb. The Fc‐silencing PGLALA mutation prevents binding of Brainshuttle‐CD20 mAbs to Fc gamma receptors and, therefore, activation of immune effector cells, and does not affect TfR1 and CD20 target binding. Uptake and transcytosis of Fc‐silent Brainshuttle‐CD20 mAbs in hTfR1‐expressing cells is also maintained when compared with Fc‐competent constructs, as demonstrated in hTfR1‐expressing cell lines. Furthermore, Fc‐silenced Brainshuttle‐CD20 mAbs display potent direct B cell death induction capacity, demonstrated by a reduction of human B cell lymphoma cells, as well as primary human B cells in WBA, tonsil‐derived cell cultures, and human CSF‐cultured PBMCs.

The additional ADCC and CDC components are considered to be the reason why Fc‐competent Brainshuttle‐CD20 mAbs demonstrate more potent B cell killing in WBAs versus Fc‐silent Brainshuttle‐CD20 mAbs, whereas in B cell lymphoma assays that assessed direct B cell killing without immune effector functions, comparable efficacy was demonstrated between the two Brainshuttle‐CD20 mAbs. In the ex vivo CSF assay, both Brainshuttle‐CD20‐PGLALA and non‐shuttled CD20‐PGLALA were effective at depleting B cells. Together, our data demonstrate that the Fc‐silencing mutations and the Brainshuttle module do not hinder CD20 target binding, and the anti‐CD20 targeting does not interfere with active brain uptake.

TfR1 is widely expressed in multiple tissues,^54^, ^55^ which can lead to systemic target‐mediated drug disposition (TMDD), as well as safety implications due to binding of the TfR1 receptor in undesired locations in the body. Data from models in this study confirm that safety concerns related to TfR1 targeting in the periphery can indeed be mitigated by Fc‐silencing, which is consistent with reported evidence of mitigation of effects on reticulocyte count in mice treated with a bivalent anti‐TfR1 antibody with eliminated Fc effector function.^39^

For the proprietary 2+1 Brainshuttle format, TfR1 binding at the C‐terminal Brainshuttle module may inhibit the effector function via steric hindrance to some extent, but not entirely for Fc‐competent constructs if the Brainshuttle‐IgG is bound to TfR1. Risk assessment for IRRs related to Brainshuttle‐CD20 binding to both TfR1 and CD20 in the periphery where the IgG target is present was critical for the progression of the program. IRRs are an inherent risk of mAb therapy, representing a major obstacle for mAb clinical development. IRR assessment and steps to reduce them are, therefore, essential. Our studies in humanized CD20 mice clearly demonstrate the mitigation of both IRRs and effects on RBC precursors with the Fc‐silenced Brainshuttle(8D3)‐CD20 mAbs. Here, hypoactivity, temperature decrease, and cytokine release, all indicative of IRRs, as well as reduced reticulocyte counts were present in mice treated with Fc‐competent Brainshuttle(8D3)‐CD20 only, demonstrating that the observed IRRs were Fc‐competence‐dependent. Interestingly, the CD20‐WT construct without the Brainshuttle(8D3) module does not cause IRRs in our transgenic mouse models, despite the fact this mAb retains the effector function and typically shows IRRs in humans. This data shows that first infusion reactions depend not only on the Fc portion of mAbs but also that the surrogate TfR1 shuttle (8D3) plays an important role in mediating the effector function in mice. Possible explanations for this may include the immobilization of opsonized B cells to the endothelial wall, where they activate effector cells, or the bridging of B cells and effector cells that also express TfR1, leading to activation via Fc gamma receptors.

To understand the possible translatability of our mouse model IRR findings to humans, we assessed Brainshuttle‐CD20 mAb variants in a human WBA. Here, proinflammatory cytokine release was demonstrated with Fc‐competent Brainshuttle‐CD20‐WT in contrast to Fc‐silenced Brainshuttle‐CD20‐PGLALA. Importantly, due to the type II killing of B cells, Fc‐silenced Brainshuttle‐hCD20‐PGLALA retained B cell killing properties. This is hypothesized to be advantageous in targeting B cells in the CNS in compartmentalized inflammation in MS, where the presence and activity of effector cells and lytic complement are uncertain or maybe reduced.^56^ Moreover, from a safety point of view, Fc‐mediated B cell killing in the CNS may induce unfavourable cytokine release. Here, we demonstrated that Brainshuttle‐CD20 mAbs improve BBB penetration to gain access to the CNS compared with a non‐shuttled CD20 mAb. We hypothesize that the improved brain penetration combined with the properties for potent Fc‐effector‐independent extravascular B cell depletion by direct B cell killing may translate to clinical efficacy for MS patients, either by preventing or slowing clinical progression. Vascular uptake and brain penetration were demonstrated in vivo via ELISA and immunohistochemistry in the mouse PK study presented herein.

In the cynomolgus macaque and transgenic mouse models, secondary lymphoid tissues containing B cell populations reflecting direct immune cell interactions were used to assess tissue‐based B cell depletion. In the huCD20 mouse single‐dose PK/PD study, neither Brainshuttle(8D3)‐CD20 mAb led to a strong B cell depletion in blood, spleen, or lymph nodes. However, B cell depletion was demonstrated in these organs in multiple‐dose studies on naïve and NP‐OVA‐treated mice, with the strongest depletion of GC class‐switched B cells. This suggests that in contrast to NHPs and the in vitro human models described above, huCD20 mice require multiple administrations of Brainshuttle(8D3)‐CD20 mAbs to achieve sufficient B cell depletion. Possible reasons for this may be the lower density of human CD20 on murine B cells in huCD20 mice, or the higher affinity of 8D3 anti‐TfR1 binder in mice relative to the 1026 binder in NHPs, leading to increased TMDD via TfR1. In the huCD20xHIGR3 mouse, it was demonstrated that Fc‐silent Brainshuttle(8D3)‐CD20 was effective in depleting GC class‐switched B cells, which are described as an important component of MS pathophysiology.^57^

The ability of Brainshuttle‐CD20 mAbs to deplete B cells was also assessed in vivo in the cynomolgus macaque, which is the most relevant preclinical model due to its close genetic, physiological, and immunological similarities to humans. In separate single‐dose PK studies, Fc‐silent Brainshuttle‐CD20 was well tolerated and demonstrated an improved safety profile compared with Fc‐competent Brainshuttle‐CD20, based on clinical signs and pathology. This confirmed our in vitro and in vivo mouse data, which indicated an increased risk for adverse effects with an Fc‐competent Brainshuttle(8D3) construct. With both Brainshuttle‐CD20 mAbs, there was a strong B cell count reduction in cynomolgus macaque blood.

## 5 CONCLUSION

The preclinical models presented herein have shown that the novel Fc‐silenced Brainshuttle‐CD20 mAb was well tolerated, with no indication of risk for IRRs, while displaying strong B cell‐killing properties. We also demonstrated TfR1‐dependent uptake of Fc‐silenced Brainshuttle(8D3)‐CD20 mAbs into the brain in huCD20 mice. Taken together, our unique Fc‐silent Brainshuttle‐CD20 mAb is hypothesized to be able to target and deplete CNS‐located B cells in MS more effectively, compared with currently available therapies. Due to the modular design of the proprietary 2+1 Brainshuttle format, the Brainshuttle module could potentially be used in combination with other therapeutic cargos, such as other mAbs or enzymes. For peripherally expressed targets, Fc‐silencing mitigates the risk of IRRs and its effects on reticulocytes, indicating a favourable safety profile for the Fc‐silenced mAbs. A Brainshuttle‐CD20 mAb with abolished Fc function has the potential to achieve higher B cell depletion efficacy in the CNS in addition to peripheral B cells, through TfR1 shuttling and direct B cell killing independent of effector functions. This contributes to the improved safety profile with regard to IRRs and haematological effects. Together, our data provide a path forward in the future development and clinical translation of TfR1‐targeting therapies for increased brain penetration. Based on these data, a Brainshuttle‐CD20 (RG6035) construct is currently in clinical trials in MS patients (NCT05704361).

## AUTHOR CONTRIBUTIONS

Vanessa L. Schumacher, Solen Pichereau, Juliana Bessa, Juergen Bachl, Sylvia Herter, Felix C. Weber, Johannes Auer, Michael Winter, Martina Stirn, Michael B. Otteneder, Kevin Brady, Anne Eichinger‐Chapelon, Nicole Clemann, Claudia Senn, Juliane Hönig, Cordula Jany, Elisa Di Lenarda, Mohammed Ullah, Niels Janssen, and Eduard Urich conceptualized the studies and study design, acquired experimental data, and were involved in the analysis and interpretation of the data. Vanessa L. Schumacher, Solen Pichereau, Juliana Bessa, and Niels Janssen wrote the initial draft. Nadine Stokar‐Regenscheit and Shanon Seger acquired experimental data and were involved in the analysis and interpretation of the data. Johannes Auer provided study materials. Anja Kipar, Adrian Roth, and Robert Mader supported the interpretation of data and writing of the manuscript. Alain C. Tissot, Christian Klein, H.‐Christian von Büdingen, and Eduard Urich conceived the concept and designed experiments. All authors provided critical commentary and revisions and reviewed and approved the final manuscript.

## CONFLICT OF INTEREST STATEMENT

V.L.S., S.H., F.J.W., N.S‐R., N.C., S.S., H.‐C.v.B., R.M., M.S., and N.J. are employees and shareholders of F. Hoffmann‐La Roche Ltd. J.Be., J.Ba., J.A., M.W., M.B.O., A.E‐C., A.R., C.S., J.H., C.J., and E.D.L. are employees of F. Hoffmann‐La Roche Ltd. S.P., K.B., A.K., M.U., and E.U. declare that they have no competing financial interests. A.C.T. and C.K. are shareholders of F. Hoffmann‐La Roche Ltd. V.L.S., A.C.T., F.J.W., N.J., C.K., and H.‐C.v.B. are named as inventors on one or more related patent applications in the name of Roche.

## ETHICS STATEMENT

Study plans and any amendments or procedures involving the care and use of animals in all experiments were reviewed and approved by the Institutional Animal Care and Use Committee of the respective institutions. All mouse studies were performed by F. Hoffmann‐La Roche (Switzerland). Pharma Research and Early Development Basel Facilities are fully accredited by the Association for Assessment and Accreditation of Laboratory Animal Care International (AAALAC), and all procedures were approved by the Cantonal Veterinary Authority of Basel‐Stadt, following strict adherence to the Swiss federal regulations on animal protection. Cynomolgus macaque studies were performed at contract research organizations in Germany and the United Kingdom, sponsored by F. Hoffmann‐La Roche; all procedures were compliant with the German Animal Welfare Act or the EU Directive 2010/63/EU, respectively.

## Supporting information



Supporting Information

Supporting Information

Supporting Information

Supporting Information

Supporting Information

Supporting Information

Supporting Information

Supporting Information

## Data Availability

All data associated with this study are present in the paper or the Supporting Information.
