# Supplementary figures and images for: Preclinical B cell depletion and safety profile of a brain‐shuttled crystallizable fragment‐silenced CD20 antibody
Source: Clin Transl Med. 2025 Mar 21;15(3):e70178. doi: 10.1002/ctm2.70178 (PMC11928292; doi:10.1002/ctm2.70178)

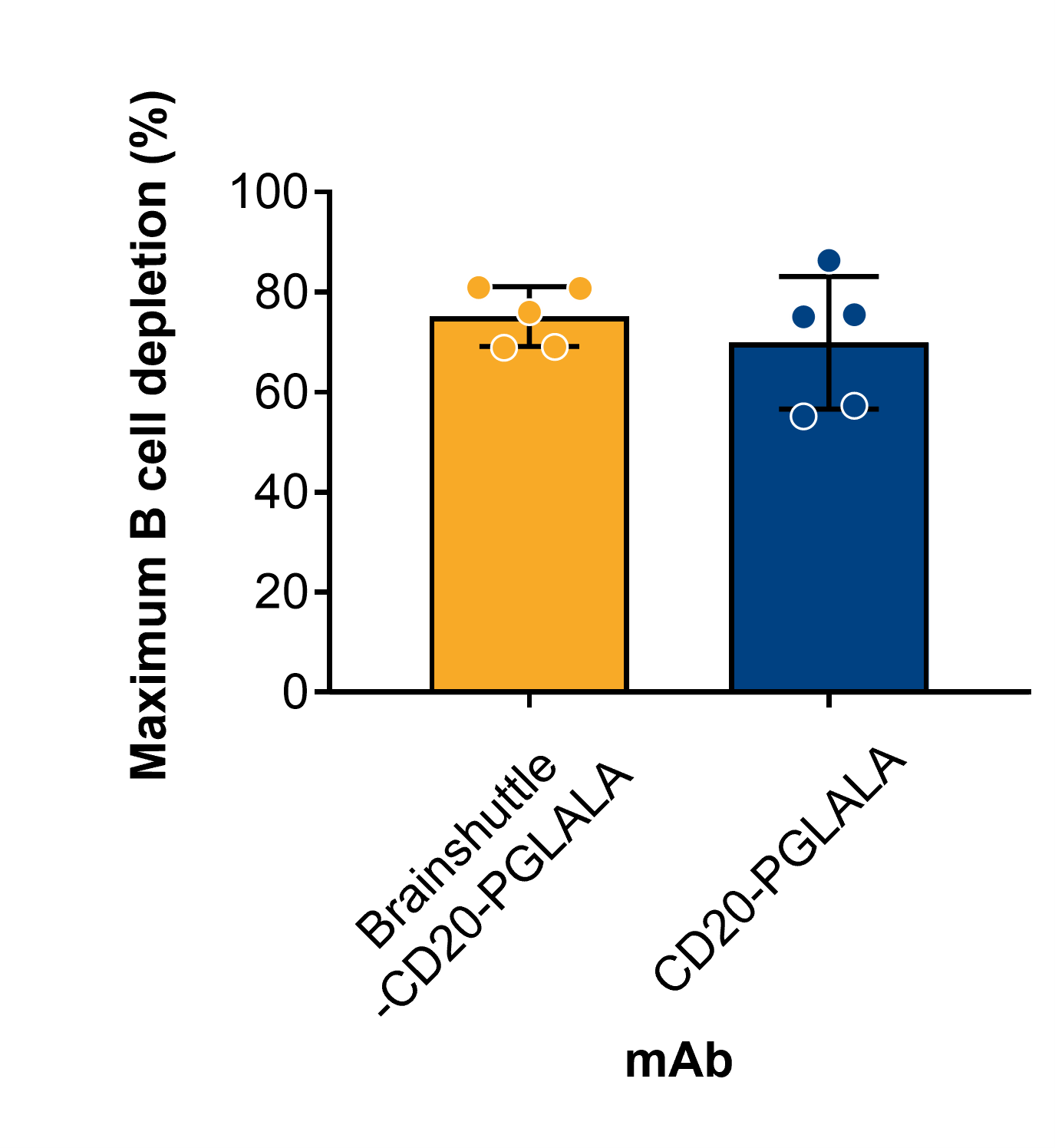

Supplement: Supplementary file 1 — Supporting Information [file CTM2-15-e70178-s003.png]

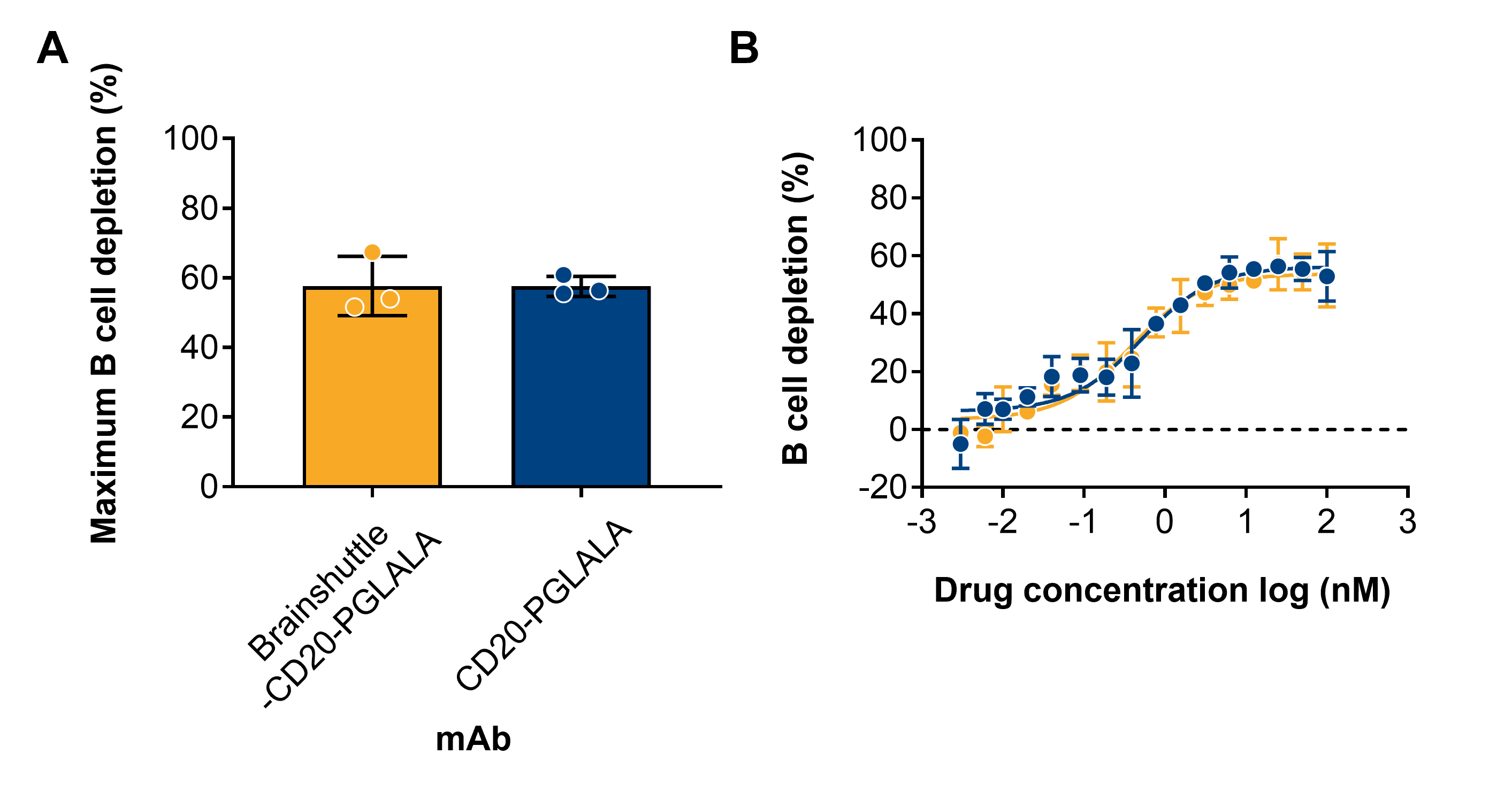

Supplement: Supplementary file 2 — Supporting Information [file CTM2-15-e70178-s005.png]

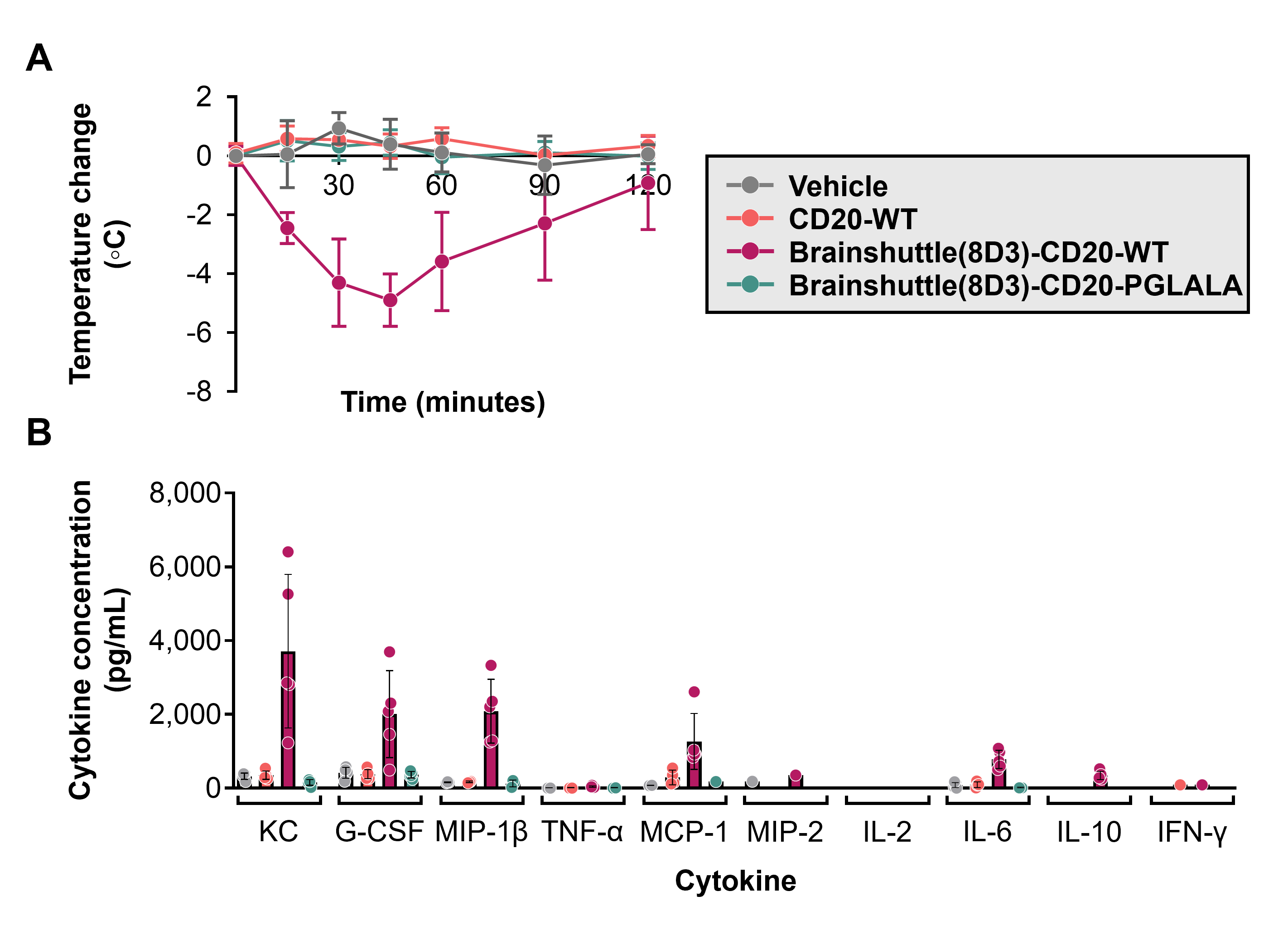

Supplement: Supplementary file 3 — Supporting Information [file CTM2-15-e70178-s002.png]

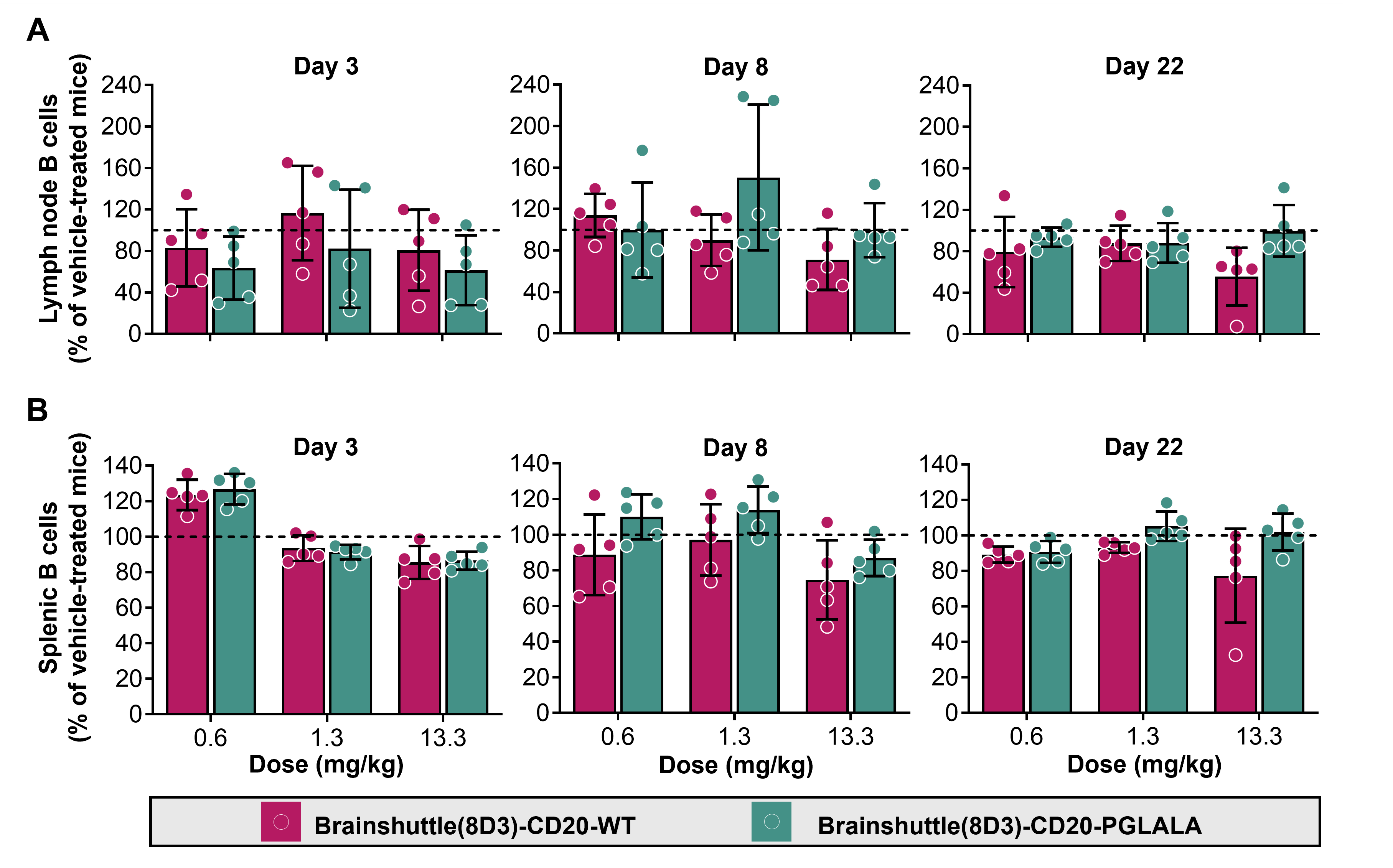

Supplement: Supplementary file 4 — Supporting Information [file CTM2-15-e70178-s008.png]

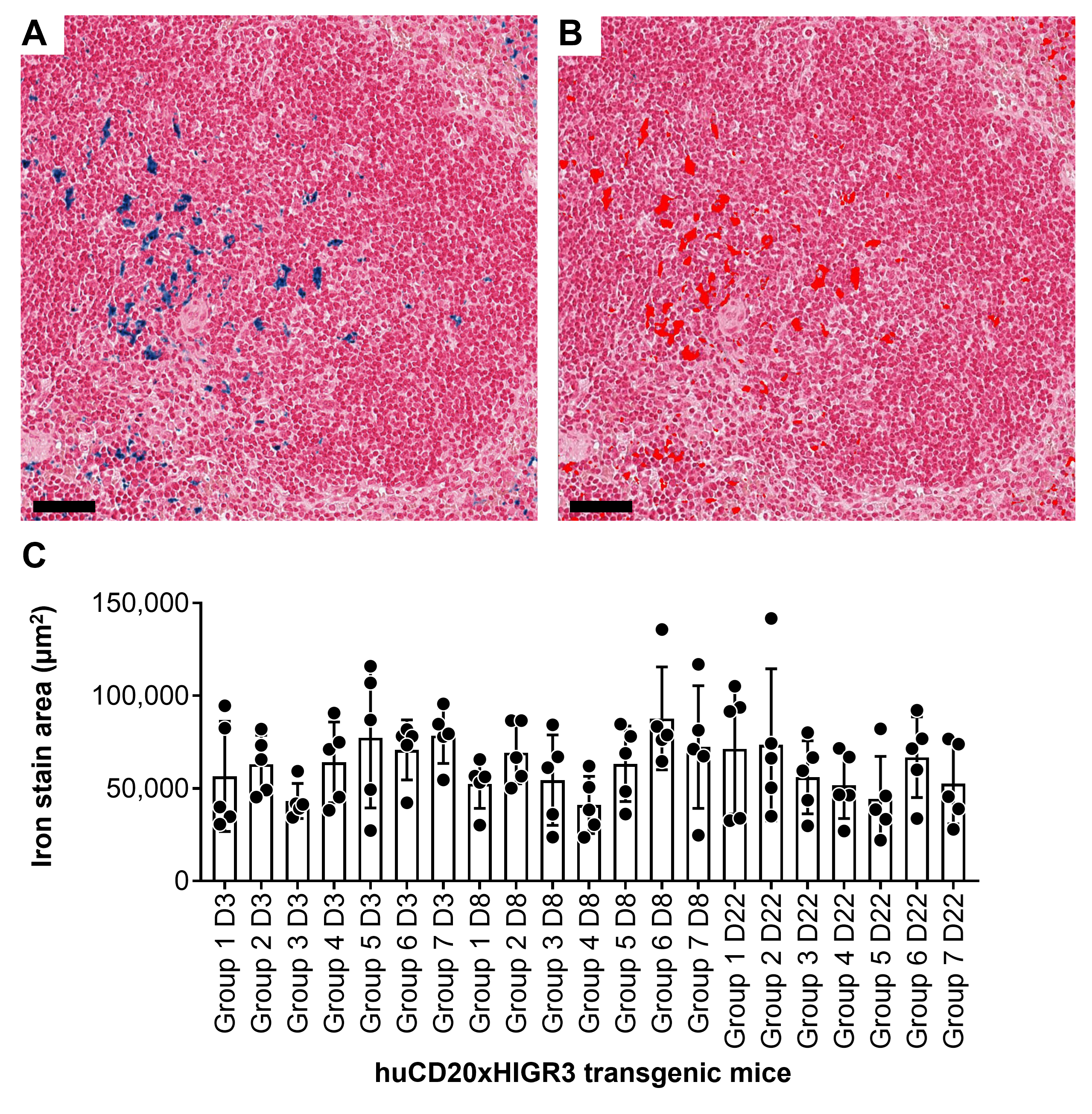

Supplement: Supplementary file 5 — Supporting Information [file CTM2-15-e70178-s006.png]

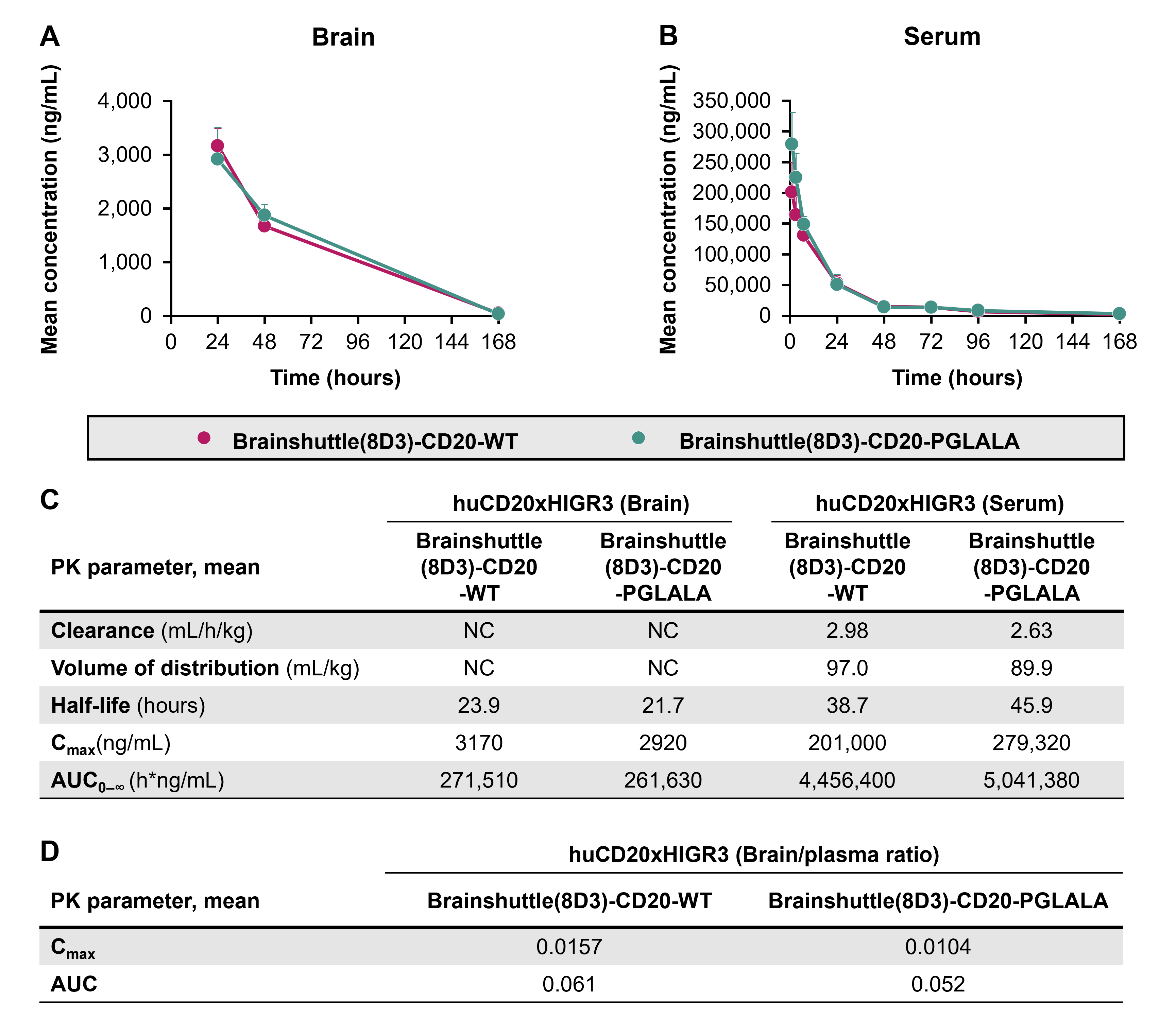

Supplement: Supplementary file 6 — Supporting Information [file CTM2-15-e70178-s007.png]

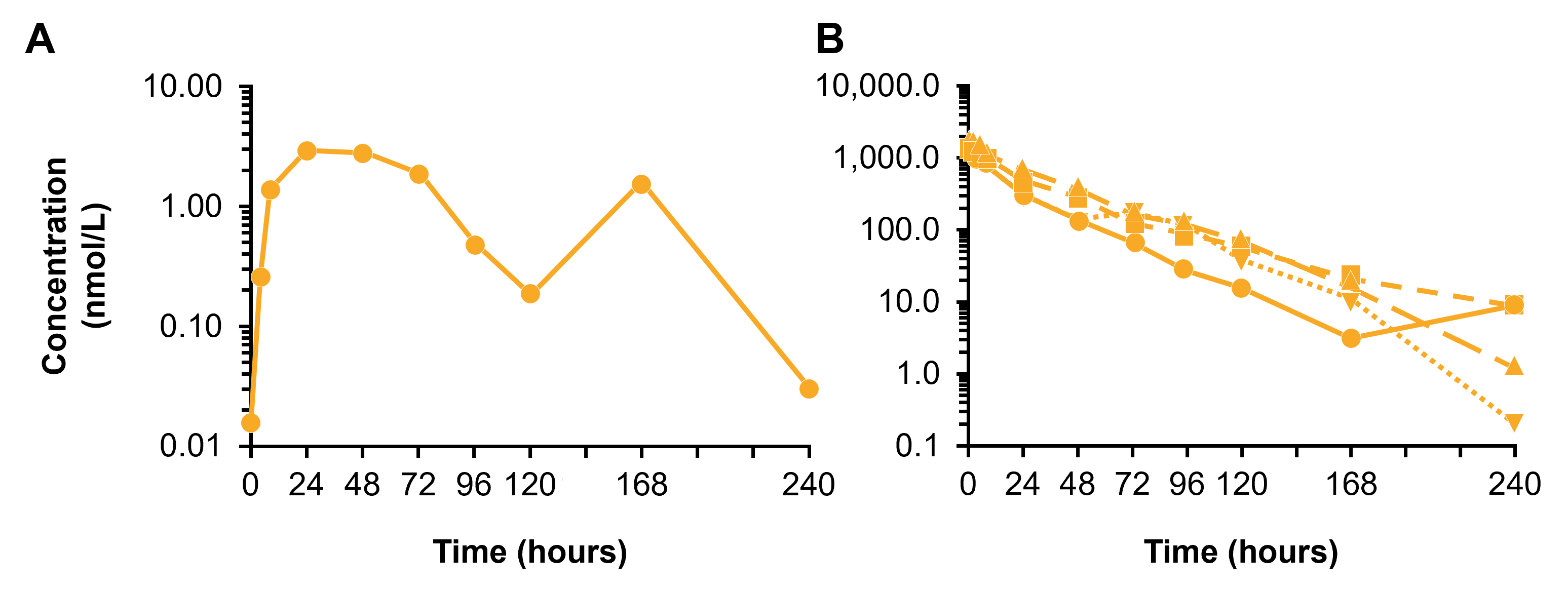

Supplement: Supplementary file 7 — Supporting Information [file CTM2-15-e70178-s004.png]
