# Supplementary material for: Preclinical B cell depletion and safety profile of a brain‐shuttled crystallizable fragment‐silenced CD20 antibody
Source: Clin Transl Med. 2025 Mar 21;15(3):e70178. doi: 10.1002/ctm2.70178 (PMC11928292; doi:10.1002/ctm2.70178)
Supplement: Supplementary file 8 — Supporting Information [file CTM2-15-e70178-s001.docx]

**Preclinical B cell depletion and safety profile of a brain-shuttled crystallizable fragment-silenced CD20 antibody**

**SUPPORTING INFORMATION**

**Authors:** Vanessa L. Schumacher^1^*, Solen Pichereau^1†^, Juliana Bessa^1^, Juergen Bachl^1^, Sylvia Herter^2^, Felix C. Weber^1^, Johannes Auer^3^, Anja Kipar^4,5^, Michael Winter^1^, Martina Stirn^1^, Michael B. Otteneder^1^, Kevin Brady^1‡^, Anne Eichinger-Chapelon^1^, Adrian Roth^6^, Nadine Stokar-Regenscheit^1^, Nicole Clemann^1^, Shanon Seger^1^, Claudia Senn^1^, Juliane Hönig^1^, Cordula Jany^3^, Elisa Di Lenarda^1^, Alain C. Tissot^3¥^, Christian Klein^2⸸^, H.-Christian von Büdingen^7^, Robert Mader^1^, Mohammed Ullah^1§^, Niels Janssen^1^, Eduard Urich^1||^

**Affiliations:**

^1^Roche Pharma Research and Early Development, Roche Innovation Center, Basel, Switzerland

^2^Roche Pharma Research and Early Development, Roche Innovation Center, Zurich, Switzerland

^3^Roche Pharma Research and Early Development, Roche Innovation Center, Penzberg, Germany

^4^Laboratory for Animal Model Pathology, Institute of Veterinary Pathology, University of Zurich, Switzerland

^5^Department of Infection Biology and Microbiomes, Institute of Infection, Veterinary and Ecological Sciences, University of Liverpool, UK

^6^Personalized Healthcare Safety, Product Development, F. Hoffmann-La Roche Ltd.

^7^Clinical Science, Neuroscience, Product Development, F. Hoffmann-La Roche Ltd.

*Corresponding author, Vanessa L. Schumacher. Email: [vanessa.schumacher@roche.com](mailto:vanessa.schumacher@roche.com)

^†^Current address: Debiopharm, Lausanne, Vaud, Switzerland

^‡^Current address: KB NBE Consulting, Charfield, Wotton-under-Edge, UK

^¥^Current address: Bayer AG, 51373 Leverkusen, Germany

⸸Current address: Curie.Bio, LLC, Boston MA02115, United States

^§^Current address: Ridgeline Discovery GmbH, Technologiepark, Hochbergerstrasse 60F, CH-4057 Basel, Switzerland

^||^Current address: Novartis Neuroscience, Basel, Switzerland

**Author Contributions:**

V.L.S., S.P., J.Be., J.Ba., S.H., F.C.W., J.A., M.W., M.S., M.B.O., K.B., A.E-C., N.C., C.S., J.H., C.J., E.D.L, M.U., N.J., and E.U. conceptualized the studies and study design, acquired experimental data, and were involved in the analysis and interpretation of the data. V.L.S., S.P., J.Be., and N.J. wrote the initial manuscript draft. N.S-R. and S.S. acquired experimental data and were involved in the analysis and interpretation of the data. J.A. provided study materials. A.K., A.R., and R.M. supported interpretation of data and writing of the manuscript. A.C.T., C.K., H.-C.v.B., and E.U. conceived the concept and designed experiments. All authors provided critical commentary and revisions and reviewed the final manuscript.

# SUPPORTING INFORMATION:

# MATERIALS AND METHODS:

## *Antibody and bispecific antibody production*

Genes for the four different antibody chains of the Brainshuttle™ and Brainshuttle(8D3) monoclonal antibodies (mAbs) were cloned into proprietary expression plasmids, which are based on pUC derivatives and harbor regulatory elements for the expression of eukaryotic genes comprising a CMV promoter, poly A signal, and transcriptional terminator. Various combinations of two genes of the four-chain complex antibody were cloned into these expression plasmids. Expi293™ or ExpiCHO-S™ cells (ThermoFisher Scientific [ThermoFisher]; A14527 and A29127, respectively) were co-transfected with the appropriate plasmid combination according to the manufacturer’s protocol and bispecific antibodies were purified from cell culture supernatants as previously described.^45^

## *CD20 binding*

Z-138 cells were maintained in RPMI1640 medium (ThermoFisher, 11875093) with 10% fetal calf serum (FCS; Gibco 16140) and 1% v/v 2 mM N-acetyl-L-alanyl-L-glutamine (GlutaMAX™; ThermoFisher, 35050061) at 0.3–0.9×10^6^ cells/mL at 37°C in a 5% CO_2_ humidified incubator. Harvested cells (98.2% viability) were pelleted by centrifugation (4 min, 400×*g*) and re-suspended in fluorescence-activated cell sorting (FACS) Buffer (phosphate buffered saline [PBS] with 2% FCS, 5 nM EDTA, and 0.25% sodium azide) at 0.56×10^6^ viable cells/mL and seeded into 96-U-bottom plates at 1×10^5^ per well. Test antibodies were added to cells with final concentrations ranging from 1000–0.0128 nM (1:5 dilution steps, in triplicate) for 30 min at 4°C. Cells were then washed with FACS Buffer and incubated with a secondary antibody (Jackson ImmunoResearch, 109-096-098; fluorescein isothiocyanate (FITC) F(ab)’2 anti-human Fcg specific; 1:20) for 30 min at 4°C. Cells were then washed twice with FACS Buffer and fixed using 2% paraformaldehyde in FACS Buffer before proceeding with FACS acquisition (BD FACS CantoII flow cytometer). FACS gating was performed using FACS Diva Software and the median fluorescence intensities were determined. Half-maximal effective concentration (EC_50_) values were calculated based on sigmoidal dose-response (variable slope) analysis using GraphPad Prism. No further statistical analysis was performed.

## *Cell death induction*

Z-138 cells were seeded in a 96-U-bottom plate at 1×10^5^ per well. Test antibodies (0.0128–1000 nM in triplicate) were incubated with cells at 37°C for 24 hours. Afterwards, cells were resuspended and centrifuged (4 minutes at 400×g), then washed with annexin V (annV) Binding Buffer (BB; 10 mM HEPES/NaOH pH 7.4, 140 mM NaCl, 2.5 mM CaCl_2_). Final cell pellets were resuspended in annV-FLUOS (Roche, 11828681001; 1:100 in annV BB) before a 15-minute incubation at room temperature with protection from light. Cells were then washed with annV BB before the addition of annV BB containing propidium iodide (PI; Merck, P4864; 1:4000). Signal acquisition was performed immediately upon addition of PI using a BD FACS CantoII flow cytometer. FACS gating was performed using BD FACS Diva Software to determine the percentages of annV/PI double positive cells. No further statistical analysis was performed.

## *TfR1-mediated cell internalization and transcytosis*

Parental Madin-Darby canine kidney II (MDCKII) cells were cultured in high-glucose Dulbecco’s Modified Eagle Medium (ThermoFisher, 41965062) supplemented with 10% FCS (Sigma-Aldrich, F4135) at 37°C in a 5% CO_2_ humidified incubator. Cells were seeded into 24-well plates at 7.5×10^4^ per well and transiently transfected 24 hours post seeding. For MDCKII transient transfection, plasmid DNA mix was prepared in Optimized Minimum Essential Medium (Opti-MEM™) reduced serum media (ThermoFisher, 31985070) at a concentration of 0.02 μg/μL, giving 0.5 μg DNA per 25 μL volume transfer per well (i.e., the total amount of DNA per 24-well plate well for transfection). Lipofectamine™ 2000 reagent (ThermoFisher, 11668027) was also diluted in Opti-MEM reduced serum media, to a concentration giving 1.5 μL Lipofectamine™ per 25 μL transfer per well. The separately prepared diluted DNA and Lipofectamine™ reagent were combined in a 1:1 ratio and incubated at room temperature for 10 minutes to allow formation of DNA/Lipofectamine™ complexes. After incubation, transfection complexes were carefully transferred into each well. Parallel mock transfections were included, where the same procedure was conducted for transfection but without the plasmid DNA.

TfR1-mediated internalization and transcytosis assays were performed 24 hours post transfection, in triplicate. The assay buffer (AB) used consisted of Hanks balance salt solution (Mg++/Ca++; ThermoFisher, 14065072) supplemented with 20 mM HEPES (ThermoFisher, #15630056), pH 7.4. All assay incubation steps were conducted at 37°C, 5% CO_2_ without shaking. Cells were rinsed twice with AB prior to initiation of the pulse phase (uptake/cell loading) with the addition 0.2 mL AB spiked with test antibodies per well. Antibodies were incubated with the cells for the indicated time points (30 or 45 minutes), depending on the experiment. At the end of the incubation, uptake was stopped with the immediate aspiration of spiked AB, followed by two rapid rinses with AB containing 0.1% bovine serum albumin (BSA), followed by a final wash with AB without BSA to remove any residual protein. The pulse step was performed in duplicate plates; one plate was immediately lysed, representing the intracellular compartment immunoglobulin (IgG) content at the start of the chase phase; time 0, and the other plate evaluated across multiple time points. Following a final rinse step (as described above), cells were incubated with pre-warmed AB (37°C) and left to initiate the chase. Samples of the AB (extracellular compartment) were then collected at the end of each time point (5, 10, and 30 minutes). At the end of the final time point, cells were rinsed three times as described above for the pulse phase, before being lysed and assayed for intracellular IgG content.

## B cell depletion in human cerebrospinal fluid (CSF)

Human peripheral blood mononuclear cells (PBMCs) and human CSF were used as a matrix for B cell depletion assays. PBMCs from five healthy donors and CSF from 12 individual donors (female and male) across all age groups, were obtained from a commercial vendor (Discovery Life Sciences). CSF samples were thawed once, pooled, aliquoted, and stored at −80°C until needed. PBMCs were thawed in RPMI 1640 medium (with 10% FCS, 1% penicillin/streptomycin [PS], and 1% non-essential amino acid [NEAA]) and equilibrated at 37°C, 5% CO_2_ for at least 2 hours. Approximately 2×10^6^ cells/mL (2×10^5^ per well) were seeded in a 96-well plate. After 2 hours of incubation, the plate was centrifuged at 300×*g* for 5 minutes at room temperature. Cells were then resuspended in 100 μL human CSF.

Treatment antibodies were diluted and added to PBMCs in CSF in the range of 0.002–100 nM for 22 hours. Cells were then pelleted and processed for fluorescence-activated cell sorting (FACS) staining with the following antibodies: CD45 (APC, F20; BD Biosciences, 560973), CD19 (PE, F20; BioLegend, 982402), and CD3 (PE/Cy7, F20; BioLegend, 300419). Cell viability was measured with the LIVE/DEAD™ Fixable Aqua Dead Cell Stain Kit (ThermoFisher, L34957), Zombie Aqua™ Fixable Viability Kit (BioLegend, 423101), and F1000 BioLegend. Samples were measured with a BD Biosciences FACSLyric™ flow cytometer using FACS Suite software in a volume-based acquisition. FACS raw data was analyzed using FlowJo software. B cell depletion was calculated using the B cell/T cell ratio of untreated control *vs*. the B cell/T cell ratio of the sample. Statistical evaluations were obtained using GraphPad Prism. Statistical significance of the difference in maximal CD19+ B cell depletion between the different mAbs was calculated using a one-way analysis of variance (multiple comparisons).

## B cell depletion in human tonsil-derived cells

Freshly resected tonsil tissue was obtained from adult donors undergoing routine tonsillectomy at Hirslanden Clinic Muenchenstein Birshof, Switzerland. Approval was given by the Ethikkommission Nordwest- und Zentralschweiz, and written patient consent was obtained for each patient-derived sample. Upon collection, tissue was stored at 4°C in Hanks’ balanced salt solution (Mg++/Ca++; ThermoFisher, 14065-072). Subsequently, tonsil tissue underwent mechanical and enzymatic digestion to obtain a cell suspension, which was directly used for B cell depletion assays. Concentration-dependent and B cell subset depletion was determined as described below.

## Total B cell depletion

B cells were transferred to 96-well U bottom plates in cultivation medium (RPMI 1640 with 10% FCS, 1% PS, and 1% NEAA at 2.5×10^5^ cells/well). Treatment antibodies were diluted and added in the range of 0.03–100 nM*.* Further experimental conditions follow B cell depletion in human CSF (described above).

## *B cell subset depletion*

Lymphocytes were identified based on size (forward scatter and granularity side scatter), as well as single-cell gating. Following a live-dead discrimination, live CD45+ lymphocytes were further gated into CD3− and CD3+ cells. CD3+ T cells were later used to normalize for B cell subset population depletion. CD19 positivity was used to identify total B cells of interest. CD19+ B cell subsets were further identified based on surface marker expression. Thereby, distinct expression of CD38 and CD10 on CD19+ B cells served as initial discriminator of three major B cell populations.

Plasma cells express the highest levels of CD38 and do not express CD10 (CD38+++/CD10−), whereas CD38-low and CD10− CD19+ B cells give rise to CD27+ memory and CD27− naïve B cells. CD38-intermediate (CD38++) and CD10+ CD19+ B cells can be further broken down to germinal center (GC) B cells, transitional B cells, and immature B cells. During transition from immature bone marrow-derived B cells to peripheral blood transitional cells, CD5 expression is upregulated. Accordingly, CD38++ CD10+ CD5+ cells have been classified as transitional B cells. Furthermore, we discriminate immature from GC B cells based on their surface IgM expression. We hereby consider IgD− CD38++ CD10+ cells as GC B cells and IgD−, but IgM+ CD38++ CD10+ as immature B cells.

## *Human whole blood assay*

Venous blood from six healthy donors was collected in vacutainer tubes containing lithium heparin as anticoagulant (Roche Medical Center, Basel, Switzerland, with written consent) and kept at room temperature until initiation of the assay (within 1–3 hours to avoid erythrocyte lysis). Antibodies were incubated with blood samples in U-bottom 96-well plates (1:40); antibody concentrations ranged from 0.01–1000 nM for Brainshuttle™-CD20-PGLALA, Brainshuttle™-CD20-WT, CD20-WT, CD20-PGLALA, and for the negative comparator cetuximab (final concentration 100 µg/mL) and the positive comparator alemtuzumab (final concentration100 µg/mL). The 1000 nM compound concentration tested covers predicted human C_max_ concentrations (maximum serum concentration). These conditions ensure optimal performance with respect to practicality and efficiency to gain at least 70 μL of plasma and sufficient cells to conduct multi-cytokine analysis. Endogenous activation of blood cells and responsiveness was assessed by including controls containing PBS, vehicle, or lipopolysaccharide. After incubation for 24 hours at 37°C, cells and plasma were separated by centrifugation at 1,800×g for 5 minutes. Plasma samples were stored at −80°C until analysis of cytokine content. Pre-tests revealed that cytokine levels did not differ between fresh and thawed samples. Determination of cytokine concentrations was performed on diluted (1:5) plasma samples. Analyte concentrations were determined by enzyme-linked immunosorbent assays (ELISAs) using multiplex cytokine chemiluminescent assay kits (Ciraplex™ Chemiluminescent Array Kit; Aushon BioSystems, 101-3EF-1-AB) with the SignaturePLUS™ imaging system and the PROarray analysis software (Aushon BioSystems).

For data points indicated as “less than” (<) the maximum indicated value was taken. For data points indicated as “greater than” (>) the minimum indicated value was taken. Data are presented as the mean cytokine measurements in supernatants from triplicate wells. Cell staining for flow cytometry was performed 24 hours following the incubation period in cell culture medium containing individual test items or controls. Cell suspensions were incubated with the antibodies of interest (**Table S1**) for 30 minutes at room temperature and washed twice with PBS to remove any non-bound antibodies. Cell samples were then analyzed on a BD Biosciences FACSCanto II flow cytometer. Flow cytometry data was analyzed using FlowJo.2, Microsoft Excel, and GraphPad Prism 7.

### Table S1. Antibodies for B cell depletion and human whole blood assay.

| **Antibody** | **Clone** | **Company**  **(Product Code)** |
| --- | --- | --- |
| **B cell subset depletion** |  |  |
| FITC Mouse Anti-Human IgM | MHM-88 | BioLegend (314506) |
| PE Mouse Anti-Human IgG | G18-145 | BD Biosciences (560951) |
| BUV395 Mouse Anti-Human CD83 | HB15e | BD Biosciences (740311) |
| PE-Cy™7 Mouse Anti-Human CD27 | O323 | ThermoFisher Scientific (25-0279-42) |
| APC-H7 Mouse Anti-Human CD10 | HI10a | BD Biosciences (655404) |
| BV786 Mouse Anti-Human CD19 | SJ25C1 | BD Biosciences (563326) |
| **Human whole blood assay** |  |  |
| PerCP-Cy™5.5 Mouse Anti-Human CD3 | UCHT1 | BD Biosciences (560835) |
| FITC Mouse Anti-Human CD14 | M5E2 | BD Biosciences (557153) |
| V450 Mouse Anti-Human CD4 | RPA-T4 | BD Biosciences (560345) |
| APC-Cy™7 Mouse Anti-Human CD8 | SK1 | BD Biosciences (348793) |
| PE-Cy™7 Mouse Anti-Human CD19 | HIB19 | BD Biosciences (560728) |
| V500 Mouse Anti-Human CD45 | HI30 | BD Biosciences (560777) |
| CD56 APC | NCAM16.2 | BD Biosciences (341027) |
| PE Mouse Anti-Human CD20 PE | L27 | BD Biosciences (347201) |
| **Control antibodies/solutions** |  |  |
| Cetuximab (Anti-EGFR Rabbit Monoclonal Chimeric IgG1) | 11E19 | Merck (ZRB04338) |
| Alemtuzumab (Anti-CD52, Humanized Rat Monoclonal IgG1) | n/a | Sanofi Genzyme |
| Lipopolysaccharide | n/a | Merck (L5886) |
| PBS | n/a | ThermoFisher Scientific (14190094) |

## *Pharmacokinetic (PK) and pharmacodynamic (PD) studies*

### Mouse single-dose PK study

Serum and brain blood tracer (DP47) levels were measured, and serum and brain samples were analyzed using two different ELISAs. Serum sampling was performed until 168 hours post dosing (based on eight time points in serum: 1, 3, 7, 24, 48, 72, 96, and 168 hours post dose [composite, 3 samples per animal]), and terminal brain sampling collection was performed at 24, 48, or 168 hours post dosing. Blood samples (0.05 mL) were collected from tail vein and heart puncture for terminal sampling time, respectively. DP47 (human IgG blood tracer for brain contamination) was administered via intravenous (IV) injection (30 mg/kg) 5 minutes prior to animal sacrifice.

For the CD20-specific ELISA method (Bioanalytical Method 1), capture antibody (mAb>M-6.28.530-IgG-Bi), positive control standards, or diluted samples and the detection antibody (mAbM.1.19.31-IgG-Dig) were successively added to streptavidin-coated microtiter plate (SA-MTP). Immobilized immune complexes were detected with a polyclonal anti-digoxigenin-horseradish peroxidase (POD) conjugate (Merck, 11633716001). Finally, the formed immobilized immune complexes were visualized by addition of ABTS™ solution (Merck, 11684302001) – a POD substrate. Color change intensity was analyzed photometrically (BioTek MTP Reader ELx808™) at 405 nm (490 nm reference wavelength) and was proportional to analyte concentrations in test samples. Before necropsy, animals were treated with IV DP47 at 30 mg/kg, 5 minutes before sacrifice. Necropsy samples (serum and brain lysates) were analyzed using an ELISA method described below. Prior to analysis, tissue samples were mechanically lysed in 800 µL of Tissue Extraction Reagent I (ThermoFisher, FNN0071) containing protease inhibitors (Merck, P8340) using the MagNA Lyser Instrument (Roche Diagnostics).

For the DP47-specific ELISA method (Bioanalytical Method 2), capture antibody (mAb>2B01-6D01-Bi), diluted calibrators, as well as diluted quality controls and samples, detection reagent (mAbM1.7.24-IgG-Dig), and anti-digoxigenin-POD were added successively to SA-MTPs. The formed immobilized immune complexes were visualized by addition of ABTS™ solution. Color change intensity was analyzed photometrically, as described above for Bioanalytic Method 1, and absorbance quantification was performed using a calibration curve with a non-linear four parameter Wiemer-Rodbard curve fitting function. Analytical sensitivity was 7.8 ng/mL in 100% serum and brain lysate with Method 1, and 11 ng/mL in 100% serum and brain lysate with Method 2.

### PK/PD study in double transgenic mice

Female humanized C57BL/6 huCD20xC57BL/6-Tg(hIg-γ1,κ,λ)ait mice (huCD20xHIGR3; Taconic Denmark) from 3–5 months of age were examined for general health and randomly assigned to groups based on body weight before study start. Clinical observations and mortality were assessed once daily before testing, twice daily on the dosing day, and once daily thereafter. Mice were treated with Brainshuttle(8D3) mAbs at 0.6, 1.3, and 13.3 mg/kg. The vehicle only group was treated with a solution containing 20 mM histidine and 140 mM NaCl at pH 6.0. Five animals per group were sacrificed post dosing at 48 hours (Day 3), 168 hours (Day 8), and 504 hours (Day 22). Selected organs were examined *in situ*, removed, checked for macroscopic abnormalities, and prepared for FACS analysis or histopathological evaluation.

Marginal zone (CD21+) and follicular B cells were also assessed in the spleen. Approximately 50 µL blood per sampling time point was collected from the tail vein for FACS analysis at pre-dose (Day 1; all 105 mice), 48 hours (Day 3; all 105 mice), 168 hours (Day 8; 70 mice), and 504 hours (Day 22; 35 mice). The following FACS antibody panel assessed was: B220, CD19, huCD20, TCRβ, CD4, CD8, CD45, CD21, CD23, IgM/IgD. Blood for hematology, exposure, anti-drug antibody (ADA) assessment, and IL-6 were terminally sampled retro-orbitally under anesthesia (ketamine/xylazine 150/9 mg/kg) shortly before exsanguination and necropsy on Days 3, 8, or 22. One sample of blood was collected into EDTA tubes to measure hematologic parameters (red blood cells [RBCs], white blood cells [WBCs], hemoglobin, hematocrit, mean corpuscular volume, mean corpuscular hemoglobin, mean corpuscular hemoglobin concentration, RBC distribution width, neutrophils, eosinophils, basophils, monocytes, lymphocytes, reticulocytes, platelets and peripheral blood smears). A 20 µL serum sample was used to determine IL-6 levels. Two 50 µL serum samples were also collected for exposure and ADA assessment.

For histopathologic assessment, inguinal lymph node, bone marrow, and spleen were collected, formalin fixed, and embedded in paraffin. Slides were prepared and stained with hematoxylin and eosin, examined by the study toxicologic pathologist and a peer reviewing toxicologic pathologist under light microscopy. For Prussian blue iron staining and assessment of iron positive percentage area using image analysis with HALO v1.2, the first step of the analysis consisted of identifying the spleen tissue *vs*. non-tissue using a random forest classifier. The identified spleen tissue was defined as region of interest (ROI). The bright-field area quantification module was used to define Prussian blue positive areas within the ROI using real time tuning to apply thresholding parameters. Area was reported as µm^2^/tissue and as percentage positive stain/tissue.

### Cynomolgus macaque single-dose studies

Two studies were performed in cynomolgus macaques. In Study 1 (Brainshuttle™-CD20-PGLALA), four females were treated with a single IV slow bolus of 10 mg/kg Brainshuttle™-CD20-PGLALA, followed by a 15-day observation period, euthanasia (sedation by intramuscular injection of ketamine hydrochloride followed by an IV sodium pentobarbitone overdose prior to exsanguination), and necropsy. Clinical observations, food consumption, body weight, body weight change, body temperature, clinical pathology (hematology, coagulation, and clinical chemistry), immunophenotyping, and cytokine evaluation were evaluated throughout the observation period, and organ weights and macroscopic/microscopic findings were also assessed.

Blood samples were collected from the cephalic or saphenous vein for hematology (0.5 mL in EDTA), clinical chemistry (2.0 mL), coagulation (1.0 mL trisodium citrate) and soluble transferrin (0.2 mL) once during the pre-dose phase, at 24 hours after dosing, and on the day of necropsy. Blood samples were taken from the cephalic or saphenous vein for cytokine analysis twice during the pre-dose phase and on Day 1 at 1- and 4-hours post dosing. CSF was collected via lumbar puncture (0.5 mL) under anesthesia, followed by application of Bepanthen^®^ aseptic wound ointment (Bayer) at four total time points per animal (timepoints included 1, 4, 8, 24, 48, 72, 96, 120, 168, 240 or 336 hours after dosing). Five minutes before euthanasia, and while the animal was sedated with ketamine, a tracer (Magnevist^®^; Bayer) was injected by IV bolus injection at a dose of 1.0 mg/kg to correct for blood contamination in brain tissue.

Cytokine levels were analyzed using Luminex multiplex kits (R&D Systems; interferon-gamma [IFNγ], tumor necrosis factor-alpha [TNFα], MCP-1, IL-6, IL-8). For immunophenotyping analysis on Day 1 (pre-dose) and at 4, 24, 72, 168, and 336 hours post dosing, blood samples (600 μL) were withdrawn from the vena cephalica antebrachii into EDTA anticoagulant. Immunophenotyping with specific mAbs comprised T cells (T helper cells and cytotoxic T cells), B cells, monocytes, and natural killer cells. Total lymphocyte counts were determined on the same day as the analysis of relative cell numbers. Absolute numbers of the lymphocyte subpopulations were determined from relative and total numbers.

In Study 2 (Brainshuttle™-CD20-WT), four females were treated with 10 mg/kg Brainshuttle™-CD20-WT with an 8-week observation period. Blood was collected from the femoral vein for cytokine analysis at pre-treatment, at 2, 24, and 72 hours post dosing, and on Days 8 and 49 (0.3 mL, EDTA). Samples were assessed with a BioRad Bio-Plex 200 reader using a custom multiplex kit for IFNγ, TNFα, MCP-1, IL-6, IL-8. Blood was also collected from the femoral vein for flow cytometry at pre-treatment, at 4, 24, and 73 hours post dosing, and on Days 5, 6, 8, 11, 13, 15, 20, 23, 28, 30, 35, 37, 42, 44, and 49 (0.5 mL sodium heparin).

For clinical pathology, blood was collected from the femoral vein at pre-treatment, Day 2, Day 8, and Day 49. For hematology, 0.5 mL target volume blood was collected into K2EDTA tubes. For coagulation, 0.9 mL was collected in 3.8% (w/v) trisodium citrate with processing to plasma. For clinical chemistry, 1.5 mL blood was collected in lithium heparin with processing to plasma. For cytokines, 0.3 mL blood was collected in K2EDTA tubes at pre-treatment, 2, 24, and 72 hours post dose, and on Days 8 and 49.

### Immunophenotyping and FACS cynomolgus macaque studies

For Study 1, the cellular antigens CD45+ and CD19+ for B lymphocyte populations were quantified using specific antibodies against marker antigens and reported as absolute counts (cells/μL of blood). WBC counts (total and absolute differential) were determined from the whole blood sample using the ADVIA^®^ 120 Hematology System (Siemens Healthineers). Total lymphocyte counts with antigen markers CD45+ CD14− CD3+ CD159α− were reported as cells/μL of blood and used for calculating absolute counts of the lymphocyte populations of interest. Change from baseline in B cells was calculated for each animal as indicated in the following equation: Percentage change in B cells = Absolute B cell count at T (hours) ×100/Absolute B cell count at T0 (hours). The SD from the mean of four animals was also calculated.

For Study 2, each mAb was labeled with a conjugate for detection: CD16 (Brilliant Violet 421), CD45 (Violet 500), CD86 (Brilliant Violet 650), CD14- (Brilliant Violet 786), CD19 (Phycoerythrin), HLA-DR (Allophycocyanine), and CD3 (Alexa Fluor 700). For the absolute counts, TruCount panel CD45 (Brilliant Violet 650), and Brilliant stain buffer were used). Samples were processed and analyzed using a three-laser LSR Fortessa X-20 cell analyzer (BD Biosciences).

# RESULTS:

## Preserved CD20 binding affinity and direct B cell killing with Fc-competent and Fc-silent Brainshuttle™-CD20

To assess B cell death induction properties in CSF, human PBMCs were incubated with Fc-silent Brainshuttle™-CD20-PGLALA and the non-shuttled CD20-PGLALA mAbs in CSF from healthy human donors. The average maximum depletion (± standard deviation [±SD]) after 22 hours incubation was 75.11% ±5.94 and 69.87% ±13.3 for Brainshuttle™-CD20-PGLALA and CD20-PGLALA, respectively (**Fig. S1**). These results indicate direct B cell depleting potency in the CSF cell culture matrix, confirming the susceptibility of central nervous system-compartmentalized B cells in CSF to the caspase-independent (non-apoptotic) B cell depletion mechanism,^28^ mediated by Brainshuttle™-CD20-PGLALA and CD20-PGLALA.


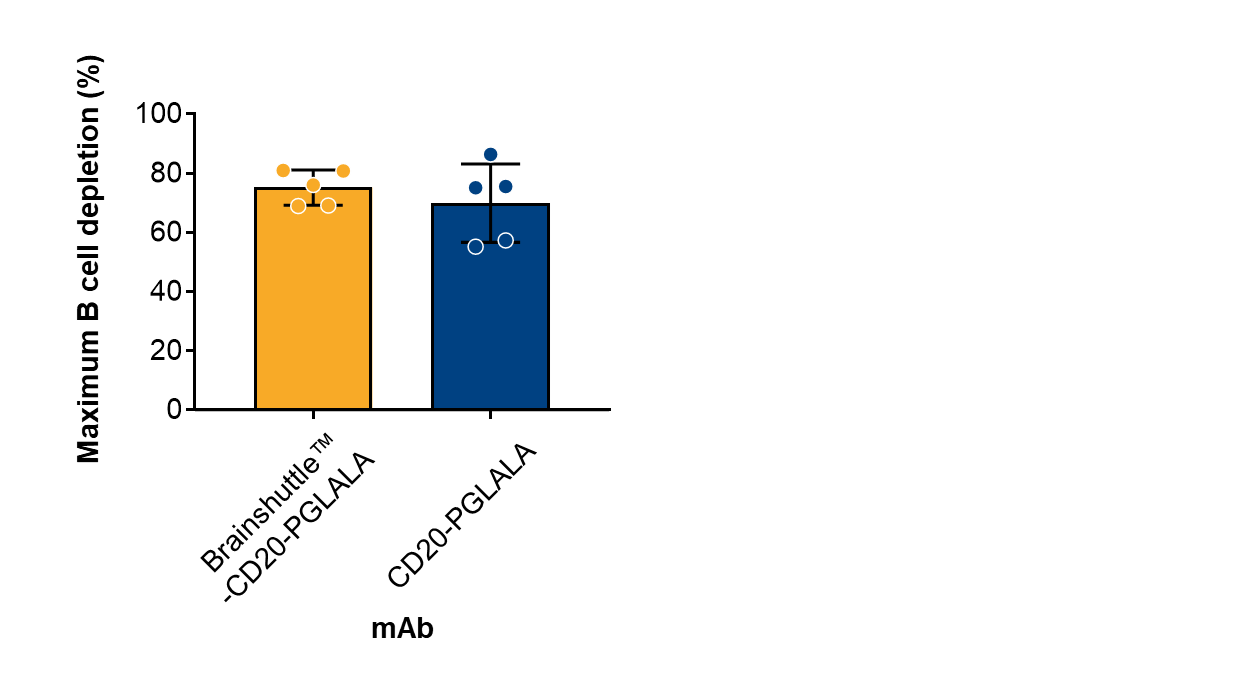


### Fig. S1. B cell depletion in human CSF with PBMCs of shuttled and non-shuttled anti-CD20 antibodies.

The average maximum depletion (±SD) after 22 hours incubation was 75.11% ±5.94 and 69.87% ±13.3 for Brainshuttle™-CD20-PGLALA and CD20-PGLALA, respectively.

CSF, cerebrospinal fluid; mAb, monoclonal antibody; PBMCs, peripheral blood mononuclear cells; PGLALA, P329GLALA mutation (for Fc silencing); SD, standard deviation.

In this PBMC-CSF assay, complement system components are absent; however, immune effector cells are still accessible. The data demonstrates that the Fc-silent Brainshuttle™-CD20-PGLALA and CD20-PGLALA mechanism of action leads to B cell depletion and does not require availability of the complement system. Upregulation of annV is typically seen in cells undergoing apoptotic processes but can be found in other forms of cell death; for example, a caspase-independent (non-apoptotic) cell depletion mechanism was recently described as a key B-cell-depleting mechanism.^28^

Brainshuttle™-CD20-PGLALA binding and B cell depletion were also assessed in an *ex vivo* autologous B cell depletion assay in human tonsil-derived cells to assess efficacy of direct cell death induction in human tonsil cell cultures across B cell subsets; B cells from tonsil tissue may better reflect the response of B cells in secondary lymphoid tissues compared with peripheral blood B cells. The present study indicates that human tonsils contain a large proportion of B cells and substantial heterogeneity of B cell subpopulations, particularly naïve B cells, tissue resident memory B cells, and immunologically active GC B cells. CD20-PGLALA induced direct CD19+ B cell death and depletion with a EC_50_ of 0.523 ±0.134 nM. The ability for direct cytotoxicity was preserved with Brainshuttle™-CD20-PGLALA (EC_50_ = 0.396 ±0.177 nM). A cytotoxicity dose effect depending on the mAb concentration was observed 22 hours after the addition of both mAbs to tonsil-derived cells. Maximum depletion was reached for both compounds in a concentration range between 25–100 nM (**Fig. S2**). There was no significant difference in maximum B cell depletion between the two mAbs at any of the tested concentrations. Brainshuttle™-CD20-PGLALA was able to reduce the amount of CD19+ human tonsil B cells by a maximum mean of 60%.


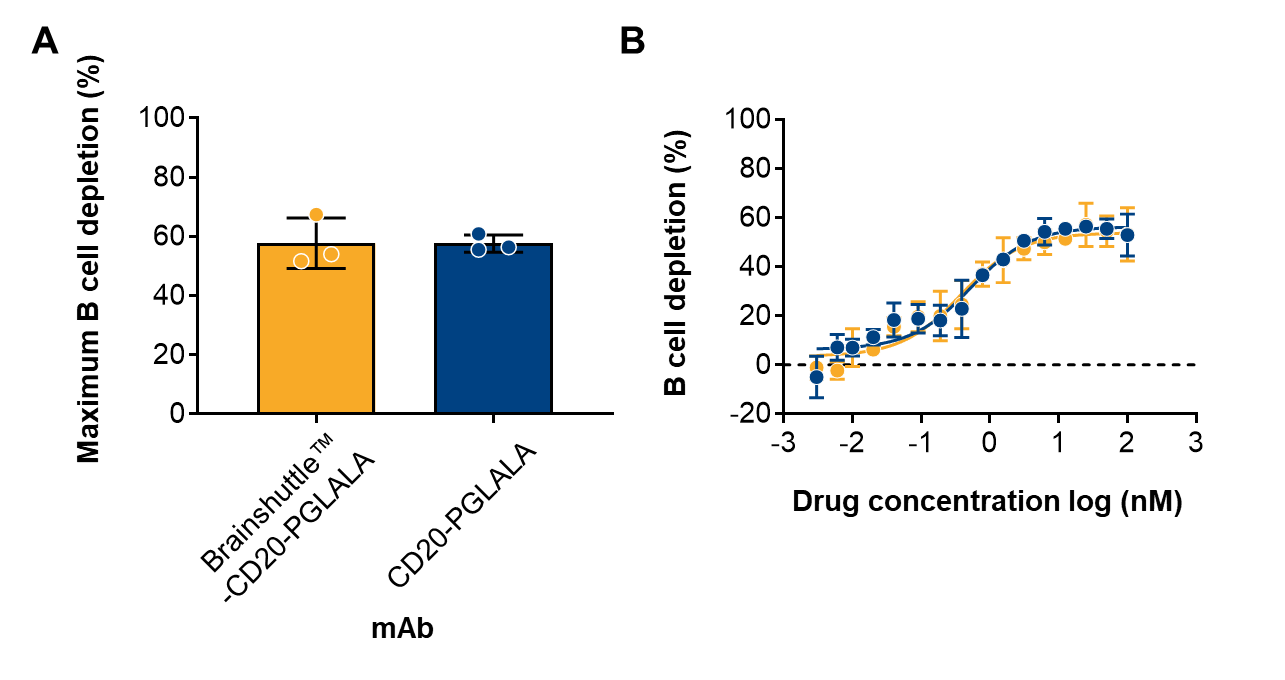


### Fig. S2. Brainshuttle™-CD20-PGLALA binding and B cell depletion in an *ex vivo* autologous human tonsil-derived B cell depletion assay.

**(A)** Average maximum CD19+ B cell depletion for the three tonsil donors. **(B)** Concentration-dependent CD19+ B cell depletion curves for three tonsil donors. Data represents mean ±SD of triplicate measurements.

mAb, monoclonal antibody; PGLALA, P329GLALA mutation (for Fc silencing); SD, standard deviation.

Three out of the four tonsil donors were used to determine the EC_50_ for CD19+ B cell depletion as well as B cell subset depletion, and there was a strikingly different cellular depletion profile between the three donors. A comparative analysis showed a correlation between maximal B cell subset depletion and surface CD20 expression, with the highest CD20 expression on transitional and the lowest CD20 expression on naïve B cells. The highest maximal depletion was observed on transitional and the lowest on naïve B cells. GC, immature, and memory B cells, which had intermediate CD20 expression levels showed intermediate maximal B cell depletion levels. The findings indicate variability in maximum depletion across B cell subsets, and that this differentiated response to Brainshuttle™-CD20-PGLALA correlates with the degree of CD20 surface expression. The findings indicate that Brainshuttle™-CD20-PGLALA is able to induce direct cell death and deplete extravascular human B cell subsets located in secondary lymphoid tissues. An explanation for incomplete depletion of B cells could be the differential expression of CD19 and CD20 on late-stage B cells, specifically plasmablasts and plasma cells that express CD19 but not CD20. Furthermore, a potential modulation of CD19 expression levels after CD20 mAb incubation could explain insufficient detection of maximal depletion.^58^ Of note, the presence of a Brainshuttle™ binding module does not negatively interfere with the B cell-depleting potency. No significant differences in EC_50_ and maximal depletion were observed between the two mAbs, proving a preserved direct B cell death induction ability of Brainshuttle™-CD20-PGLALA despite Brainshuttle™-mediated human transferrin receptor 1 (hTfR1) co-binding.

## Reduced risk of infusion-related reactions with Fc-silent Brainshuttle(8D3)-CD20

As with huCD20xHIGR3 mice, body temperature data for huCD20 mice demonstrate a clear differentiation between treatments; all animals treated with Brainshuttle(8D3)-CD20-WT experienced an acute temperature drop of approximately 2–5°C, indicative of an acute infusion-related reaction, followed by a quick recovery, while animals treated with CD20-WT or Brainshuttle(8D3)-hCD20-PGLALA experienced no drop in body temperature (**Fig. S3A**). As in huCD20xHIGR3 transgenic mice, Brainshuttle(8D3)-CD20-WT induced strong serum release of keratinocyte-derived cytokine (KC), granulocyte colony stimulating factor (G-CSF), macrophage inflammatory protein-1β (MIP-1β), monocyte chemoattractant protein-1 (MCP-1) and a moderate interleukin-6 (IL-6) release compared to Brainshuttle(8D3)-CD20-PGLALA in huCD20 mice; other cytokines were largely unaffected (**Fig. S3B**).


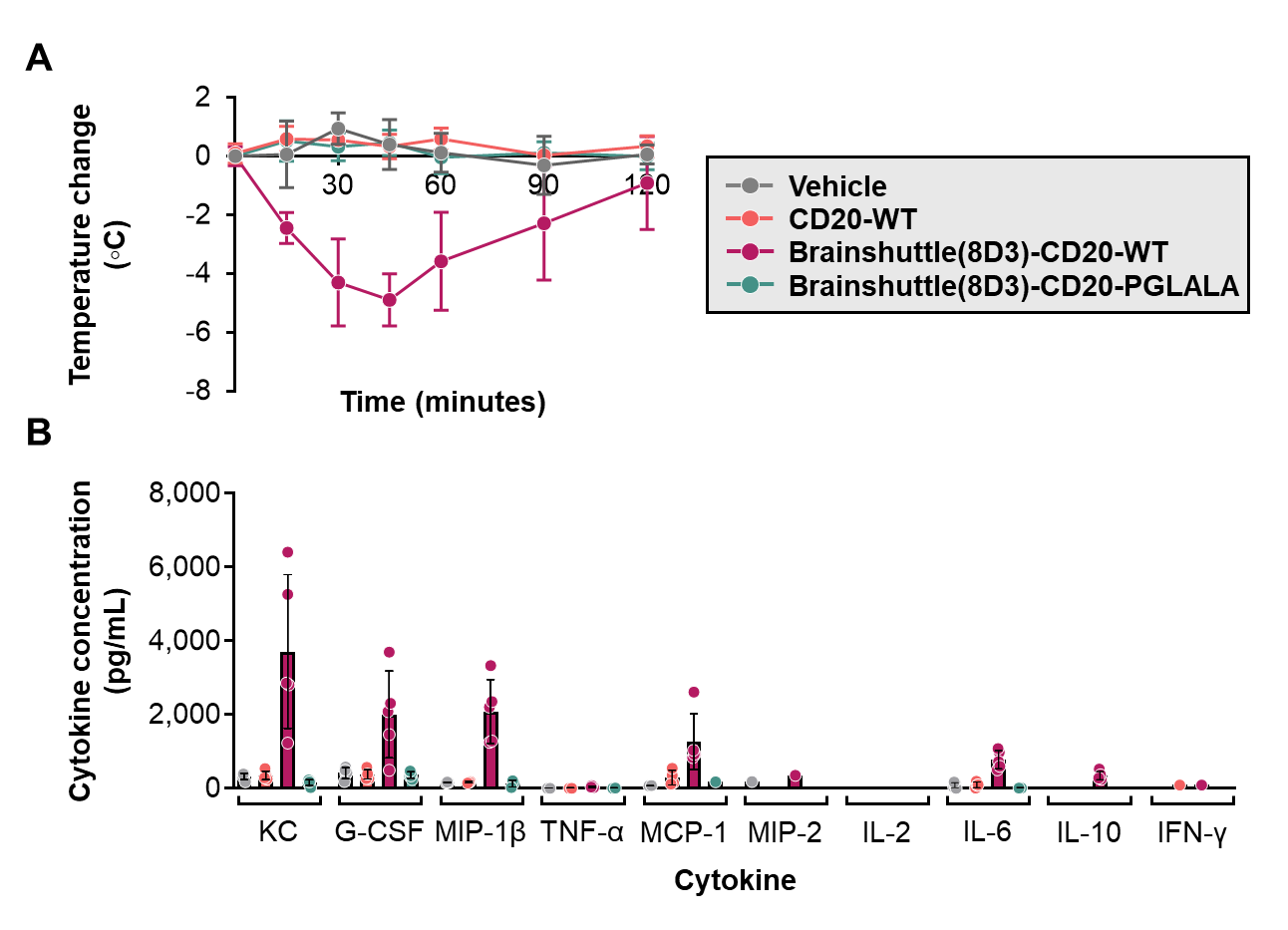


### Fig. S3. Effect of Fc silencing on body temperature and cytokine levels in huCD20 mice.

(**A**) Effects of Brainshuttle(8D3)-CD20 mAbs on body temperature in huCD20 transgenic mice induced following IV administration of Brainshuttle(8D3)-CD20 mAbs at 10 mg/kg compared to non-shuttled CD20-WT and IV administration of vehicle only. (**B**) Cytokine levels measured 2 hours after administration of Brainshuttle(8D3)-CD20 mAbs. Data points represent mean ±SD in n=5 huCD20 mice per group.

G-CSF, granulocyte colony stimulating factor; huCD20, humanized CD20; IFN-γ, interferon-gamma; IL, interleukin; IV, intravenous; KC, keratinocyte-derived cytokine; mAbs, monoclonal antibodies; MCP, monocyte chemoattractant protein; MIP, macrophage inflammatory protein; PGLALA, P329GLALA mutation (Fc-silent); SD, standard deviation; TNF-α, tumor necrosis factor-alpha; WT, wild-type (Fc-competent).

## Fc-silencing mitigates effects on reticulocytes in transgenic mice while maintaining B cell depletion properties

In huCD20xHIGR3 mice, the most pronounced reduction of B cell numbers was observed on Day 3 (~50%; **Fig. S4A**), which may not necessarily reflect B cell depletion, but rather egress to peripheral blood. In the spleen, there was no observed effect of Brainshuttle(8D3)-CD20-PGLALA, and only a minimal and non-dose dependent was observed with Brainshuttle(8D3)-CD20-WT (**Fig. S4B**). Histological findings in the spleen consisted of an increase in extramedullary hematopoiesis on Days 3 and 8 with return to normal on Day 22 with both Brainshuttle(8D3)-CD20 mAbs. No difference in iron accumulation was observed in the spleen between treated and control animals (**Fig. S5**).


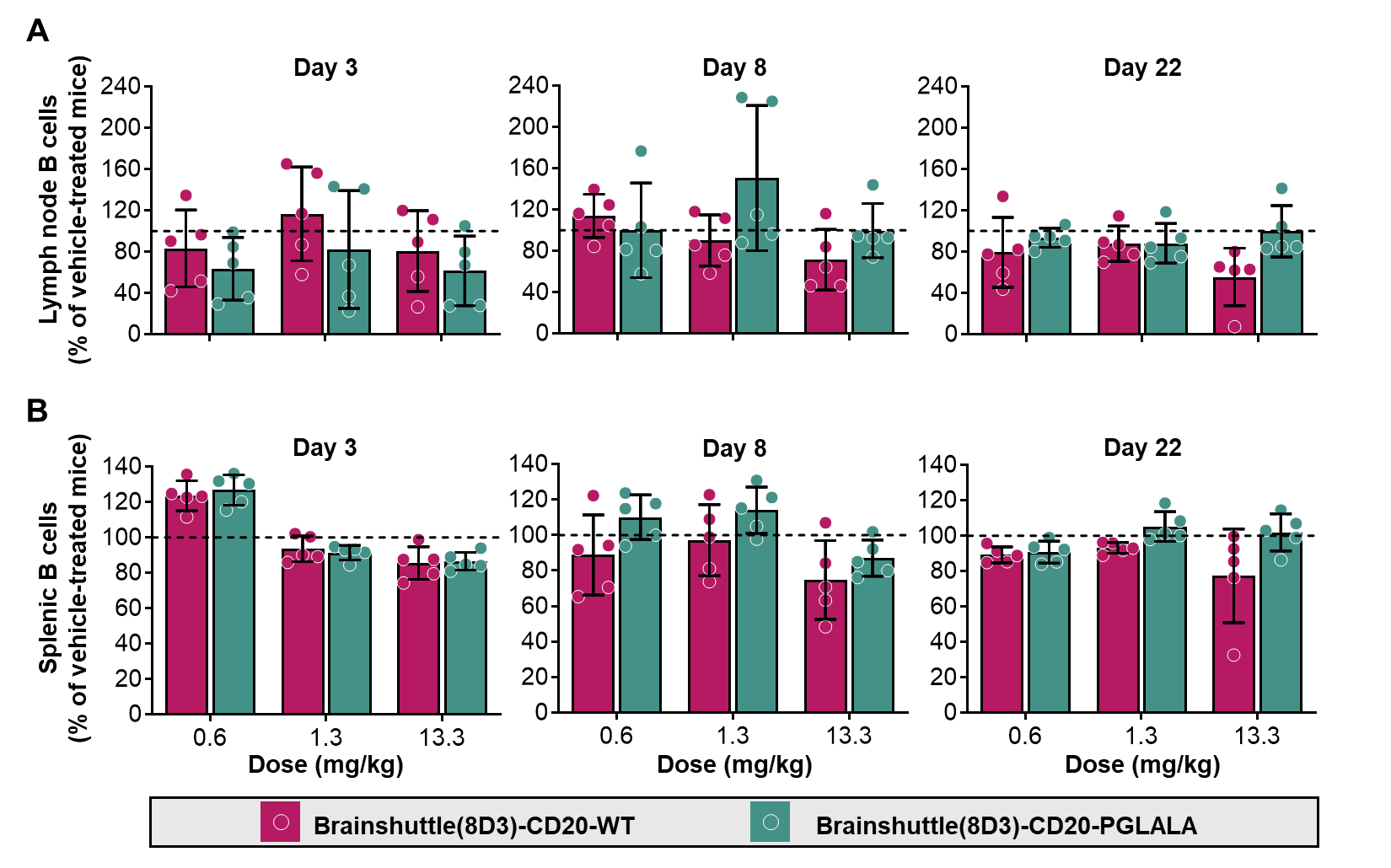


### Fig. S4. B cell depletion in response to treatment with Brainshuttle(8D3)-CD20 mAbs by FACS analysis.

The percentage of remaining B220+ B cells in lymph nodes (**A**) and spleen (**B**) were calculated by setting the frequencies of B220+ cells in vehicle-treated animals as 100% (dashed line). Data represents mean ±SD measurements from n=5 huCD20xHIGR3 mice per group.

FACS, fluorescence-activated cell sorting; huCD20xHIGR3, C57BL/6 huCD20xC57BL/6-Tg(hIg-γ1,κ,λ)ait mice; mAbs, monoclonal antibodies; PGLALA, P329GLALA mutation (Fc-silent); SD, standard deviation; WT, wild-type (Fc-competent).


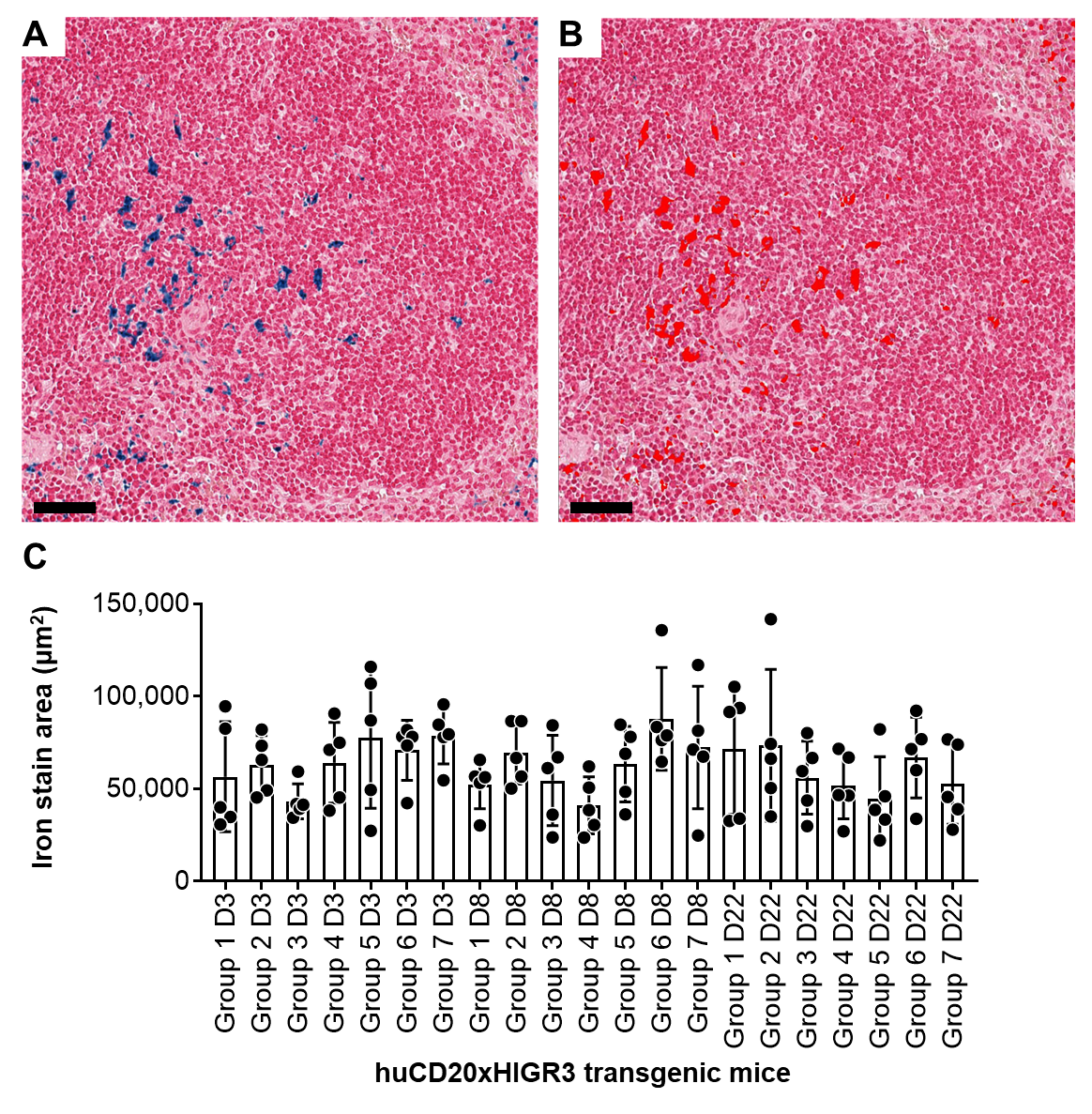


### Fig. S5. Quantification of iron staining in the spleen of huCD20xHIGR3 transgenic mice.

Prussian blue iron staining in the spleen of transgenic mice demonstrates no significant difference in iron storage between Brainshuttle(8D3)-CD20-WT (**A**) and Brainshuttle-CD20-PGLALA (**B**) treatment groups. Images taken at 20× magnification. Scale bars = 50 µm. (**C**) For each slide, image analysis quantification of positive iron-stained area was normalized to tissue area in the seven groups of huCD20 mice at Day 3, 8, and 22.

huCD20xHIGR3, C57BL/6 huCD20xC57BL/6-Tg(hIg-γ1,κ,λ)ait mice; PGLALA, P329GLALA mutation (Fc-silent); WT, wild-type (Fc-competent).

## PK exposure and brain penetration in huCD20xHIGR3 transgenic mice

Twelve females were given an IV administration of 13.3 mg/kg Brainshuttle(8D3)-CD20 mAbs for PK assessment. Serum samples were collected at 1, 3, 7, 24, 48, 72, 96 and 168 hours post administration. Four animals were sacrificed at three timepoints (24, 48, and 168 hours) to assess brain exposure. At 5 minutes prior to brain harvest (before transcardial perfusion using a peristaltic pump for 6 minutes), an additional dose of the DP47 tracer (30 mg/kg) was administered to account for any blood contamination. The mean concentration of the Brainshuttle(8D3)-CD20 mAbs in both brain and serum were similar (**Fig. S6A** and **Fig. S6B**, respectively). PK parameters for both mAbs in both brain and serum were also similar (**Fig. S6C**). Brain-to-serum ratios are shown in **Fig. S6D**.


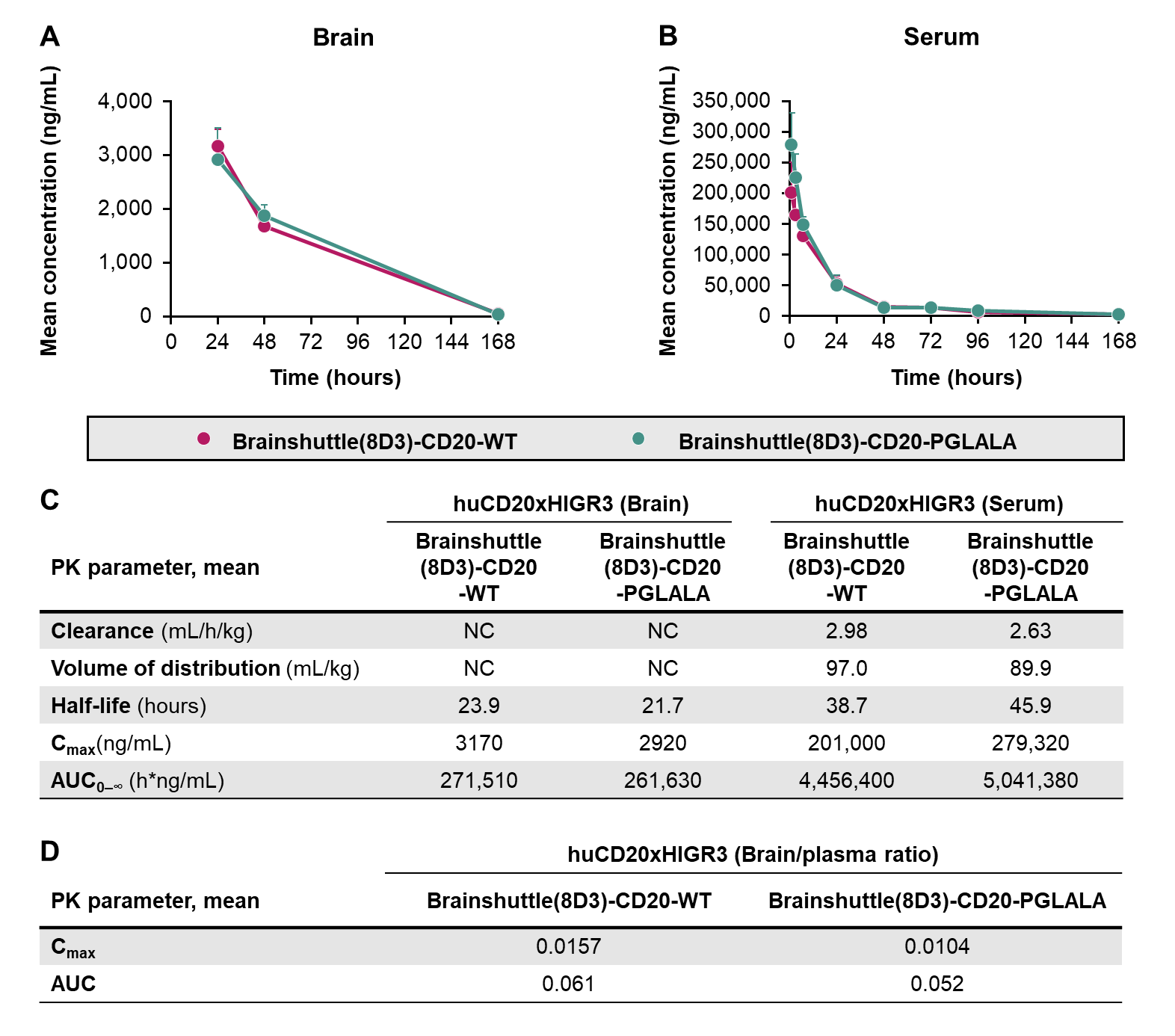


### Fig. S6. PK of Brainshuttle(8D3)-CD20 mAbs in huCD20xHIGR3 transgenic mice.

Mean concentration of Brainshuttle(8D3)-CD20 mAbs in brain (**A**) and serum (**B**). Mean PK parameters in both brain and serum (**C**) and brain-to-serum ratios (**D**). Data represents mean +SD of n=4 animals.

AUC, area under the curve; C_max_, maximum concentration; huCD20xHIGR3, C57BL/6 huCD20xC57BL/6-Tg(hIg-γ1,κ,λ)ait mice; NC, not calculated; PGLALA, P329GLALA mutation (Fc-silent); PK, pharmacokinetic; WT, wild-type (Fc-competent).

## Fc-silenced Brainshuttle™-CD20 depletes B cells in cynomolgus macaques with a favorable safety profile

In cynomolgus macaques treated with Brainshuttle™-CD20 WT on Day 2, there were mild-to-moderate increases in absolute neutrophil counts and monocyte counts reflected in increases in total white blood cell counts. Clinical chemistry changes consisted of mildly decreased phosphorus concentrations on Day 2, and minimal-to-mild decreased triglyceride and cholesterol concentrations on Day 2 and/or Day 8. Additionally, minimal increases in fibrinogen, C-reactive protein, haptoglobin, and ferritin concentrations, and minimal decreases in iron concentrations were observed on Day 2 and/or Day 8. Together with the changes in absolute neutrophil and monocyte concentrations, these changes were indicative of an acute phase response. On Day 49, the observed changes in hematology, coagulation, and clinical chemistry (with the exception of triglyceride changes at 1 mg/kg) were no longer present, indicating recovery. These findings are indicative of normal regenerative erythropoiesis in response to multiple blood collections, consistent with the observed minimal-to-moderate drop in red cell mass parameters (RBC count, hemoglobin, and hematocrit levels) and the increase in reticulocyte counts.

Composite CSF concentrations of Brainshuttle™-CD20-PGLALA following a single IV bolus of 10 mg/kg are shown in **Fig. S7A**. Plasma concentrations for each of the four non-human primates (NHPs) are shown in **Fig. S7B**.


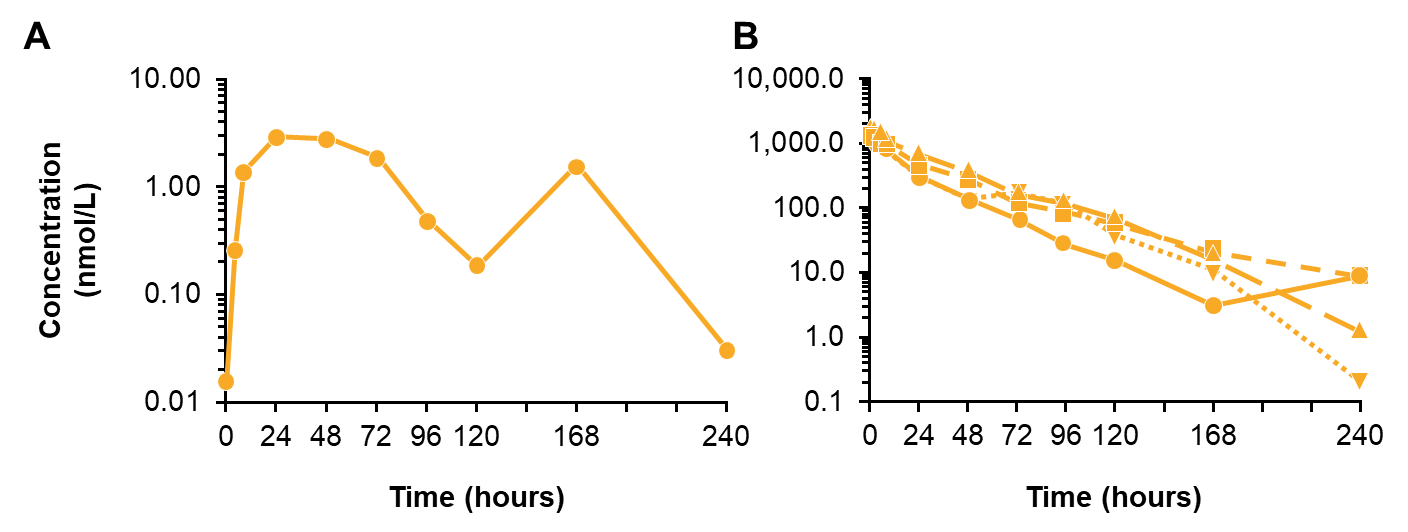


### Fig. S7. CSF and plasma levels of Brainshuttle™-CD20-PGLALA in cynomolgus macaques.

Composite CSF concentration (from four animals) of Brainshuttle™-CD20-PGLALA following a single IV bolus of 10 mg/kg ranged from 0.183 to 4.71 nmol/L in the 24–168-hour time window (**A**). Plasma Brainshuttle™-CD20-PGLALA concentrations in four animals following a single IV bolus of 10 mg/kg (**B**).

CSF, cerebrospinal fluid; IV, intravenous; PGLALA, P329GLALA mutation (Fc-silent).

# References:

28. Klein C, Lammens A, Schäfer W, et al. Epitope interactions of monoclonal antibodies targeting CD20 and their relationship to functional properties. *MAbs*. 2013;5(1):22-33. doi:10.4161/mabs.22771

45. Schaefer W, Regula JT, Bähner M, et al. Immunoglobulin domain crossover as a generic approach for the production of bispecific IgG antibodies. *Proc Natl Acad Sci USA*. 2011;108(27):11187. doi:10.1073/pnas.1019002108

58. Jones JD, Hamilton BJ, Rigby WF, Rituximab mediates loss of CD19 on B cells in the absence of cell death. *Arthritis Rheum*. 2012;64(10):3111-3118. doi: 10.1002/art.34560
